# Reduced Intensity Conditioning Followed by Allogeneic Hematopoietic Stem Cell Transplantation Is a Good Choice for Acute Myeloid Leukemia and Myelodysplastic Syndrome: A Meta-Analysis of Randomized Controlled Trials

**DOI:** 10.3389/fonc.2021.708727

**Published:** 2021-10-07

**Authors:** Yanzhi Song, Zhichao Yin, Jie Ding, Tong Wu

**Affiliations:** ^1^ Bone Marrow Transplantation, Beijing Boren Hospital, Beijing, China; ^2^ School of Medicine, Johns Hopkins University, Baltimore, MD, United States

**Keywords:** reduced intensity conditioning (RIC), acute myeloid leukemia, myelodysplastic syndrome, overall survival, non-relapse mortality (NRM)

## Abstract

**Background:**

Reduced intensity conditioning (RIC) before allogeneic hematopoietic stem cell transplantation (allo-HSCT) has been reported to have the same overall survival (OS) as myeloablative conditioning (MAC) for patients with acute myeloid leukemia (AML) in complete remission (CR) and myelodysplastic syndrome (MDS). However, results from different studies are conflicting. Therefore, we conducted a systematic review and meta-analysis guided by PRISMA 2009 to confirm the efficacy and safety of RIC vs. MAC for AML in CR and MDS.

**Methods:**

We search PubMed, Web of Science, Embase, Cochrane central, clinical trial registries and related websites, major conference proceedings, and field-related journals from January 1, 1980, to July 1, 2020, for studies comparing RIC with MAC before the first allo-HSCT in patients with AML in CR or MDS. Only randomized controlled trials (RCTs) were included. OS was the primary endpoint and generic inverse variance method was used to combine hazard ratio (HR) and 95% CI.

**Results:**

We retrieved 7,770 records. Six RCTs with 1,413 participants (711 in RIC, 702 in MAC) were included. RIC had the same OS (HR = 0.95, 95% CI 0.64–1.4, *p* = 0.80) and cumulative incidence of relapse as MAC (HR = 1.18, 95% CI 0.88–1.59, *p* = 0.28). Furthermore, RIC significantly reduced non-relapse mortality more than total body irradiation/busulfan-based MAC (HR = 0.53, 95% CI 0.36–0.80, *p* = 0.002) and had similar long-term OS and graft failure as MAC.

**Conclusion:**

RIC conditioning regimens are recommended as an adequate option of preparative treatment before allo-HSCT for patients with AML in CR or MDS.

**Systematic Review Registration:**

https://www.crd.york.ac.uk/PROSPERO/display_record.php?RecordID=185436.

## Introduction

Allogeneic hematopoietic stem cell transplantation (allo-HSCT) has the lowest risk of relapse than any other treatment for acute myeloid leukemia (AML) or myelodysplastic syndrome (MDS) (1). However, allo-HSCT, like traditional myeloablative conditioning (MAC) regimens, has been associated with a high risk of serious adverse events and high non-relapse mortality (NRM) (2). Over the past three decades, the development of less toxic and more tolerable pre-transplantation regimens—the reduced intensity conditioning (RIC) regimen—has thus become the focus of clinical research (3). Specifically, the RIC regimens consisted of less than 8 Gray (Gy) of total body irradiation (TBI), less than 8 mg/kg PO of busulfan (Bu), or intravenous equivalent dose or other medications with high-powered immuno-suppressive effect but with less tissue toxicity to replace TBI or Bu along with fludarabine (Flu) to replace cyclophosphamide (Cy) (3). RIC reduces tissue injury and consequently reduces the incidences of acute graft versus host disease (aGVHD) and other complications but maintains graft versus leukemia effect to prevent leukemia relapse (3). Some non-randomized controlled studies reported that RIC reduced NRM but increased disease relapse, generally resulting in the same overall survival (OS) as MAC (4–6). However, these observational studies lack the benefit of random allocation, which is important to balance the baseline characteristics of patients among different treatment arms, especially to control for confounding by indication bias. Recently, several high-quality randomized controlled trials (RCTs) compared RIC with MAC for fit patients with AML in complete remission (CR) and MDS, but the results were not consistent (7–12).

The number of patients receiving RIC is rapidly increasing. In the United States, RIC accounts for more than 50% of all allo-HSCTs (13). Except for AML and MDS, there have been no prospective studies comparing RIC with MAC for other hematologic malignancies. Therefore, we undertook this systematic review (SR) and meta-analysis to clarify the efficacy and safety of RIC versus MAC for AML in CR and for MDS.

## Methods

This meta-analysis was guided by PRISMA 2009 guidelines (**Supplement 1**). The meta-analysis protocol is registered on PROSPERO with the ID of CRD42020185436.

We included only RCTs compared RIC with MAC before first allo-HSCT in patients with AML in CR or MDS, as defined by 2008 World Health Organization (14) (recruitment began after 2008) and French–American–British criteria (recruitment began before 2008). We did not restrict for age, sex, race, recruitment period, complicated diseases, or languages and allowed any aGVHD prophylaxis regimens except *in vitro* T-cell depletion. Median follow-up time should be more than 1 year.

The primary endpoint was OS, whereas the secondary endpoints were leukemia-free survival (LFS), cumulative incidences of relapse (CIR), NRM, aGVHD, and chronic (c) GVHD. Survival data were evaluated from the first day after stem cell transfusion until the first occurred event and the longest follow-up data were used. Glucksberg (15), International Bone Marrow Transplant Registry grading systems (16), and Seattle criteria (17) were used to grade aGVHD and cGVHD. Incidences of III–IV aGVHD, extensive cGVHD, graft failure (GF), overall organ toxicity, oral mucositis, specific organ toxicities, and reported infection were safety endpoints.

We electronically searched databases and hand-searched field-related articles between January 1, 1980, and July 1, 2020. **Supplement 2** showed the detailed searching strategy. Cochrane highly sensitive search filters were used for identifying RCTs in Medline and Embase (18).

YS and ZY independently screened retrieved records, extracted data of the characteristics of included studies according to **Table 1** and **Supplement 3**, and used Cochrane Collaboration-recommended tool to assess quality of included studies (**Table 2** and **Supplement 3**) (19). Only studies in the low-risk group were included and disagreement was resolved by discussion through YS, ZY, and JD. We also contacted authors if additional information was required.

**Table 1:** Demographic characteristics of included studies.

Studies		Beelen et al. (8)		Bornhäuser et al. (9)	Kröger et al. (10)		MC-FludT.14/L Trial I (7)		Ringdén et al. (11)	Scott et al. (12)	
Recruitment period		Jan 25^th^, 2013-November 16^th^, 2016		Nov 15^th^, 2004-Dec 31^st^, 2009	May 2004-December 2012		Nov 24^th^, 2008–Sep 26^th^, 2012		N/R	June 2^nd^, 2011-April 10^th^, 2014	
Number of participants	RIC	240		99	65		168		18	137	
	MAC	220		96	64		152		19	135	
Median age (range), years	RIC	61.0 (56.5–64.0)		44 (18–60)	51 (22-63)		58.0 (54.0-63.0)		46 (26-61)	54.8 (21.9-65.9)	
	MAC	60.0 (55.0–65.0)		45 (18–60)	50 (19-64)		59.0 (53.0-63.0)		45(22-58)	54.8 (21.9-66)	
Diagnosis (number)	RIC	AML in CR (138); MDS (102)		AML in CR (99)	MDS (61); sAML in CR (4)		AML in CR (109); MDS (43)		AML in CR (14); CML in CP1 (4)	AML in CR (110); MDS (27)	
	MAC	AML in CR (155); MDS (65)		AML in CR (96)	MDS (54); sAML in CR (8); missing (2)		AML in CR (130); MDS (38)		AML in CR (15); CML in CP1 (4)	AML in CR (108); MDS (27)	
Number of high risk	RIC	AML in CR: 43; MDS: 55		22	7		N/R		3	71	
	MAC	AML in CR: 63; MDS: 36		26	9		N/R		3	54	
Donor source (number)	RIC	MRD, MUD		MRD, MUD	MRD, MUD		MRD, MUD		MRD, MUD	MRD, RUD, MUD	
	MAC	MRD, MUD		MRD, MUD	MRD, MUD		MRD, MUD		MRD, MUD	MRD, RUD, MUD	
Performance status before HSCT	RIC	HCT-CI Score >2, number (percentage)	140 (58%)	Participants have adequate renal, cardiac, pulmonary, and neurological function.	ECOG (number)	0 (21), 1 (29), 2 (3), 3 (2), Missing (10)	HCT-CI Score, Median (Q1, Q3)	3.0 (2.0, 5.0)	Patients who would tolerate MAC without advanced diseases.	HCT–CI Score, (number)	0 (40), 1–2 (52), ≥3 (44)
	MAC	HCT-CI Score >2, number (percentage)	131 (60%)	Participants have adequate renal, cardiac, pulmonary, and neurological function.	ECOG (number)	0 (18), 1 (32), 2 (3), 3 (0), Missing (11)	HCT-CI Score, Median (Q1, Q3)	3.0 (1.0, 4.0)	Patients who would tolerate MAC without advanced diseases	HCT–CI Score, (number)	0 (46), 1–2 (45), ≥3 (42)
Conditioning regimen	RIC	Bu 6.4 mg/kg intravenously + Flu 150 mg/m^2^		TBI 8 Gy + Flu 120 mg/m²	Bu 8 mg/kg + Flu 150 mg/m^2^		Bu 6.4 mg/kg intravenously + Flu 150 mg/m^2^		Bu 8mg/kg + Flu 150–180 mg/m^2^	Bu 8 mg/kg + Flu (120–180 mg/m^2^); Flu (120-180 mg/m^2^) + Mel (≤150 mg/m^2^)	
	MAC	Treosulfan 30 g/m² + Flu 150 mg/m²		TBI 12 Gy + Cy 120 mg/kg	Bu 16 mg/kg + Cy 120 mg/kg		Treosulfan 42 g/m² + Flu 150 mg/m²		Bu 16 mg/kg + Cy 120 mg/kg	Bu 16 mg/kg or TBI (12-14.2 Gy) + Flu (120-180 mg/m^2^ or Cy 120mg/kg)	
Median follow-up time, months	RIC	17.4		119	72		12		40.8	50	
	MAC	15.4		119	75		12		62.4	50	
GVHD prophylaxis	RIC	CsA/MTX		CsA/MTX	CsA/MTX		CsA/MTX		CsA/MTX	CNI/MMF, CNI/MTX, Tac/Siro	
	MAC	CsA/MTX		CsA/MTX	CsA/MTX		CsA/MTX		CsA/MTX	CNI/MMF, CNI/MTX, Tac/Siro	
Withdrawn/all randomized (%)		16/476 (3.48)		0/195 (0)	0/129 (0)		10/330 (3)		0/37 (0)	0/272 (0)	

N/R, not reported; RIC, reduced intensity conditioning; MAC, myeloablative conditioning; AML, acute myeloid leukemia; CR, complete remission; MDS, myelodysplastic syndrome; sAML, secondary AML; CML, chronic myeloid leukemia; CP1, the first chronic phase; MRD, matched related donor; MUD, matched unrelated donor; RUD, related mismatched donor; HCT–CI, hematopoietic cell transplantation-comorbidity index; ECOG, Eastern Cooperative Oncology Group; Q1, the first quartile; Q3, the third quartile; Bu, busulfan; Flu, fludarabine; TBI, total body irradiation; Gy, Gray; Mel, melphalan; Cy cyclophosphamide; CsA, cyclosporine; MTX, methotrexate; CNI, calcineurin inhibitor; MMF, mycophenolate mofetil; Tac, tacrolimus; Siro, sirolimus.

**Table 2:** Quality assessment of included studies.

Studies	Random sequence generation (selection bias)	Allocation concealment (selection bias)	Blinding of participants and personnel (performance bias) All outcomes	Blinding of outcome assessment (detection bias) All outcomes	Incomplete outcome data (attrition bias) All outcomes	Selective reporting (reporting bias)	Other bias
Beelen et al. (8)	Low risk	Low risk	Low risk	Low risk	Low risk	Low risk	Unclear risk
Bornhäuser et al. (9)	Low risk	Low risk	Low risk	Low risk	Low risk	Low risk	Low risk
Kröger et al. (10)	Low risk	Low risk	Low risk	Low risk	Low risk	Low risk	Low risk
MC-FludT.14/L Trial I (7)	Low risk	Low risk	Low risk	Low risk	Low risk	Low risk	Unclear risk
Ringdén et al. (11)	Low risk	Low risk	Low risk	Low risk	Low risk	Low risk	Low risk
Scott et al. (12)	Low risk	Low risk	Low risk	Low risk	Low risk	Low risk	Low risk

We used Cochrane Collaboration-recommended tool to assess the quality of included studies (19). The studies were classified into low-risk and high-risk groups. Studies reporting sufficient information to show low risk of bias in the sequence generation and allocation concealment were stratified into the low-risk group; otherwise, they were stratified into the high-risk group. Studies with high risk in any other domains were stratified into the high-risk group, too. Funnel plots and meta-regression would be used to assess publication bias.

Revman software (Version 5.3; Copenhagen: The Nordic Cochrane Centre, The Cochrane Collaboration, 2012) was used. Hazard ratio (HR) and its 95% confidence interval (CI) were combined in the meta-analyses of OS, CIR, LFS, NRM, aGVHD, and cGVHD endpoints with generic inverse variance method (20). Statistics of log HR and variance were calculated according to Parmar et al. (21), Mantel-Haenszel (22), and DerSimonian–Laird (23) methods calculating relative risk (RR) or odds ratio (OR), and 95% CIs were used to combine dichotomous data. Two-sided *p* < 0.05 was considered significant. Heterogeneity was calculated with *Q* test and *I* ^2^ statistics. Fixed effect model was used if heterogeneity was not significant (*p* > 0.10 and *I* ^2^ < 50%). Random effects model was used if heterogeneity was significant (*p* ≤ 0.10 and/or *I* ^2^ ≥ 50%). Because treosulfan was less toxic than TBI/Bu (8, 24), we predefined three subgroups that were named RIC vs. TBI/Bu-based MAC, RIC vs. treosulfan 30 g/m^2^-based MAC, and RIC vs. treosulfan 42 g/m^2^-based MAC, respectively. In addition, in NRM and aGVHD meta-analyses, we only combined HR of every subgroup but the total HR of all included studies was not combined. Except for NRM and aGVHD, both the HR in the three subgroups and all included studies were combined. Sensitivity analyses removing included studies were used to evaluate whether quality of studies and clinical characteristics influenced results. Funnel plot and meta-regression were planned to detect publication bias.

Quality of evidence on main endpoints were evaluated with the “GRADE evidence profiles” table (25).

## Results

**Figure 1:**
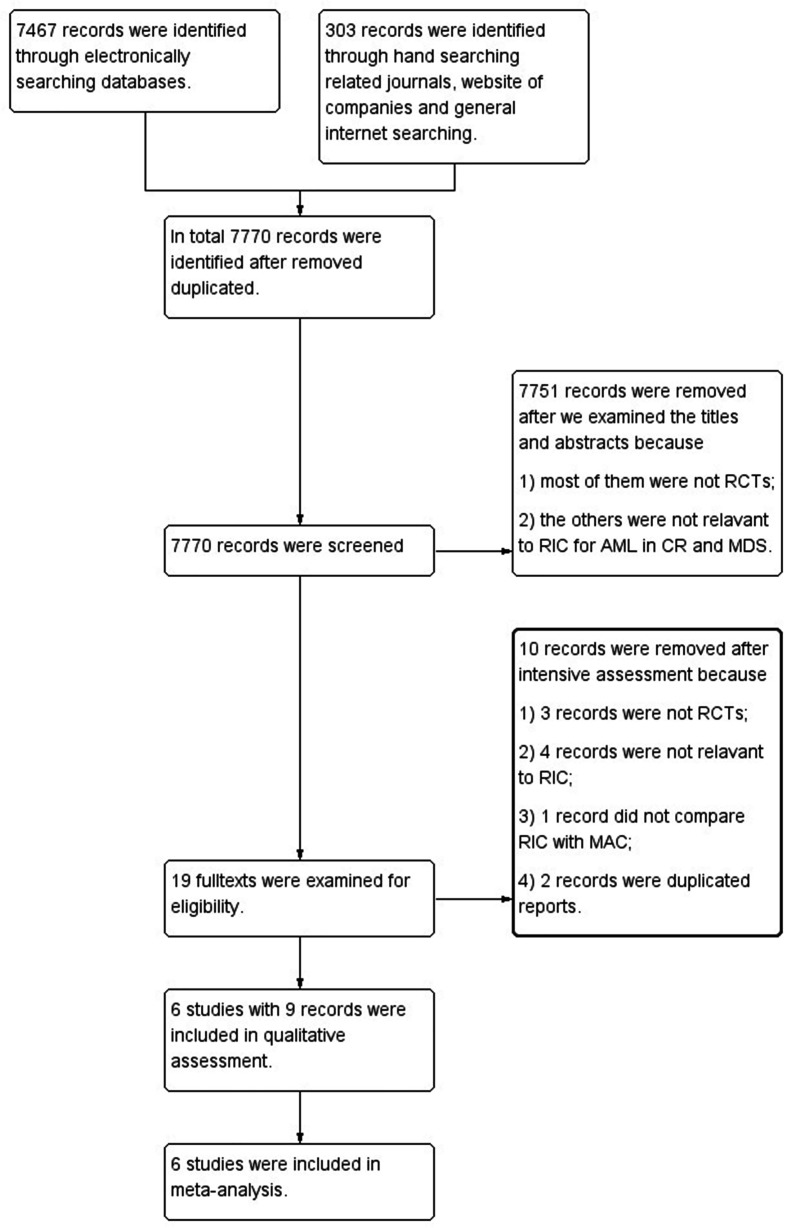
Flow diagram of screening studies for inclusion in systematic review. AML, acute myeloid leukemia; CR, complete remission; MDS, myelodysplastic syndrome; RCTs, randomized controlled trials; RIC, reduced intensity conditioning; MAC, myeloablative conditioning.

Our search retrieved 7,770 references. After reviewing the titles and abstracts, 7,751 records were excluded for the reason that they were not relevant to RIC for AML in CR and MDS or not RCTs. After further examining full texts of the remaining 19 records, we excluded 10 references that were not RCT studies, not relevant to RIC, not compared with MAC regimens, or duplicated reports. In the end, we included 6 RCTs reported in 9 references into meta-analyses. All authors agreed to include Bornhäuser et al. (9), Kröger et al. (10), Ringdén et al. (11), Scott et al. (12), Beelen et al. (8) and MC-FludT.14/L Trial I studies (7) (**Figure 1**). Studies of Bornhäuser et al. (9), Kröger et al. (10), Ringdén et al. (11), and Scott et al. (12) reported the long-term follow up data (11, 26–28).

The six included studies with 1,413 participants (711 in the RIC group and 702 in the MAC group) all focused on the efficacy and safety of RIC compared with MAC, followed by allo-HSCT for AML in CR and MDS. Four studies focused on RIC vs. TBI/Bu-based MAC, whereas two studies focused on RIC vs. treosulfan-based MAC regimens. Studies used peripheral stem cell and bone marrow as stem cell sources. Donors included matched related, mismatched related, and matched unrelated donors. The demographic characteristics of the two treatment arms were similar in the included studies and are shown in **Table 1**. All included studies displayed low risk of bias. Details of quality assessment of the included studies are shown in **Table 2** and **Supplement 3**. All studies used the intention-to-treat method to analyze OS, CIR, and LFS. There was no selective reporting in all the included studies. Because funnel plots and meta-regression should only be used with more than 10 studies, we did not use them to detect publication bias in our analysis (29).

**Figure 2:**
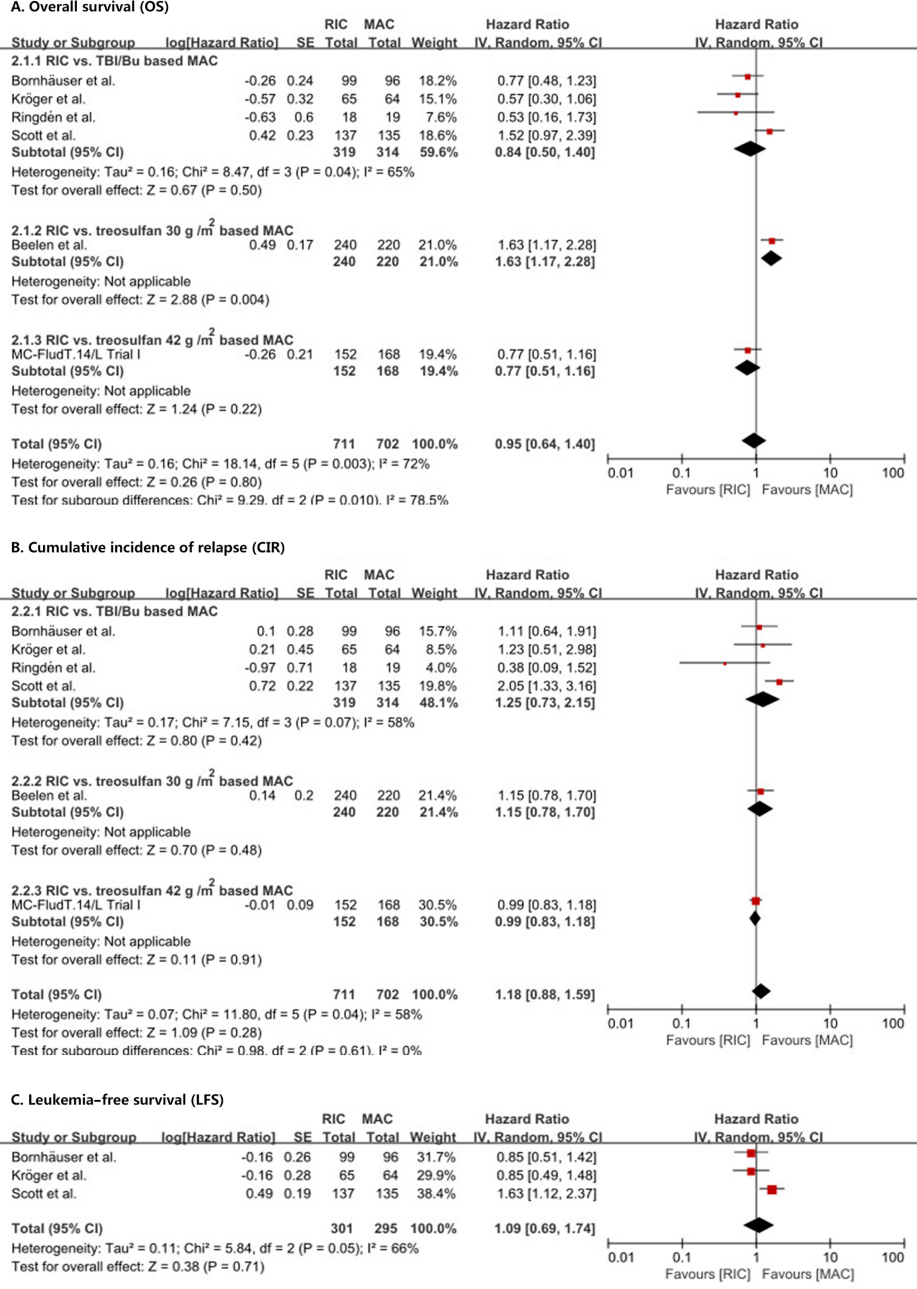
Results of meta-analyses of OS, CIR, and LFS endpoints. The forest plots showed that RIC had the same OS **(A)**, CIR **(B)**, and LFS **(C)** as MAC. RIC, reduced intensity conditioning; MAC, myeloablative conditioning; TBI, total body irradiation; Bu, busulfan.

**Figure 3:**
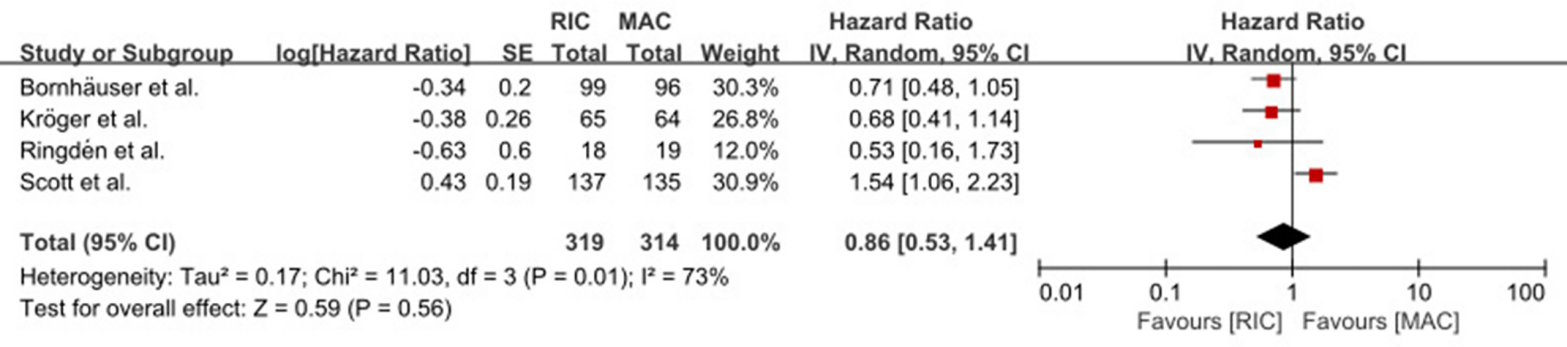
Result of meta-analysis of long-term OS data. The forest plot showed that RIC had the same long-term OS as TBI/Bu-based MAC. OS, overall survival; RIC, reduced intensity conditioning; MAC, myeloablative conditioning; TBI, total body irradiation; Bu, busulfan.

OS was not statistically (*p* = 0.80) different between RIC and MAC (HR = 0.95, 95% CI 0.64–1.4). Heterogeneity of the meta-analysis was significant (*p* = 0.003, *I* ^2^ = 72%) (**Figure 2A**). The result was also similar in the RIC vs. TBI/Bu-based MAC subgroup analysis (HR = 0.84, 95% CI 0.5–1.4, *p* = 0.50) with significant (*p* = 0.04) heterogeneity (*I* ^2^ = 65%). However, in the RIC vs. treosulfan 30 g/m^2^-based MAC subgroup analysis, RIC was significantly (*p* = 0.004) lower than treosulfan-based MAC conditioning regimen (HR = 1.63, 95% CI 1.17–2.28). The combined long-term follow-up data also showed no difference between RIC and MAC (HR = 0.86, 95% CI 0.53–1.41, *p* = 0.56) with significant (*p* = 0.01) heterogeneity (*I* ^2^ = 73%) (**Figure 3**).

We did not find a significant (*p* = 0.28) difference in CIR (HR = 1.18, 95% CI 0.88–1.59) between RIC and MAC (**Figure 2B**) and in CIR in the three subgroup analyses. Heterogeneity in the meta-analysis and in the RIC vs. TBI/Bu-based MAC subgroup was significant. Bornhäuser et al. (9), Kröger et al. (10), and Scott et al. (12) reported LFS, the combined result showed RIC had similar LFS to MAC (HR = 1.09, 95% CI 0.69–1.74, *p* = 0.71) with significant (*p* = 0.05) heterogeneity (*I* ^2^ = 66%) (**Figure 2C**).

**Figure 4:**
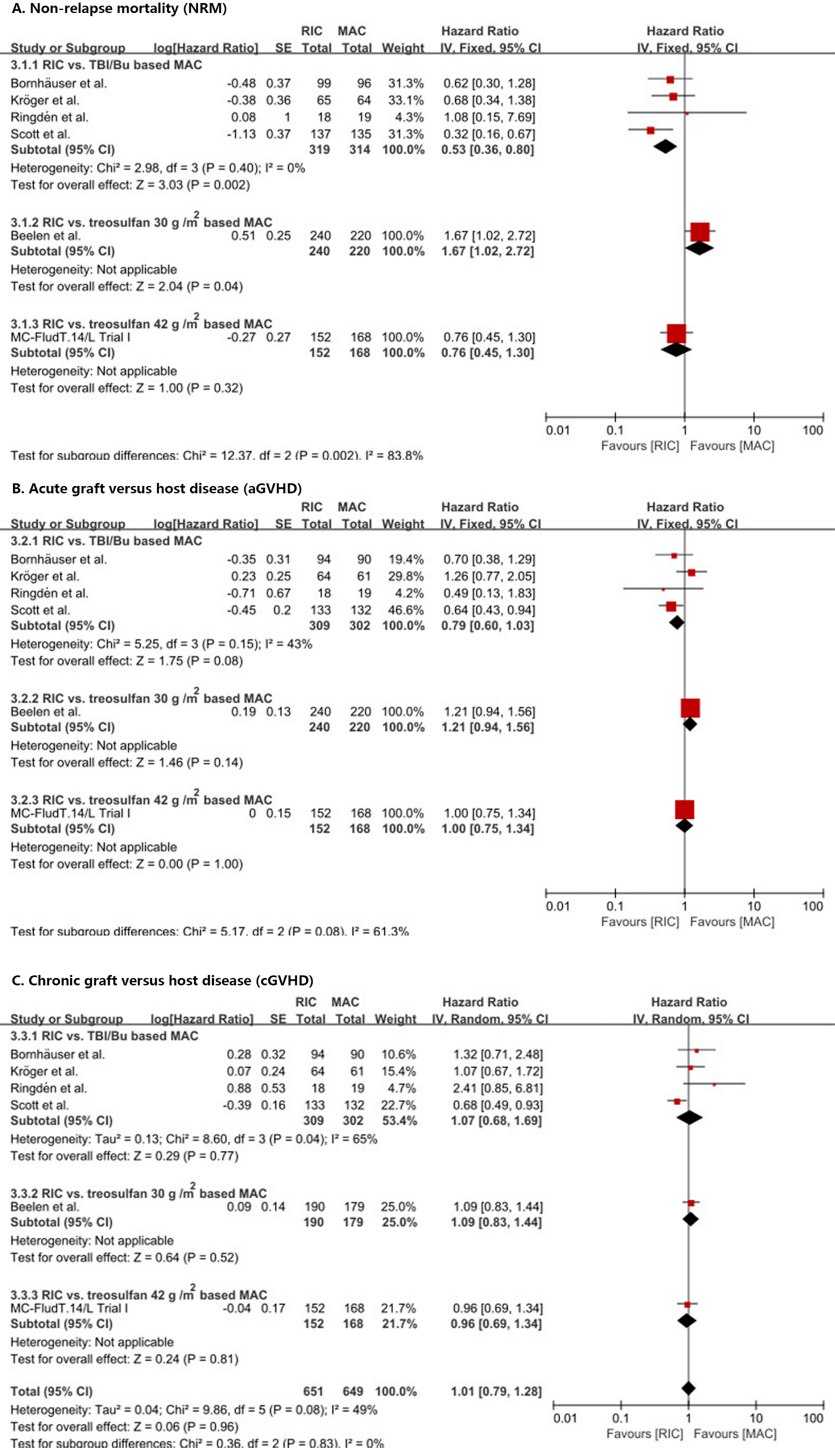
Results of meta-analyses of NRM, aGVHD, and cGVHD endpoints. The forest plots showed that RIC significantly decreased NRM than TBI/Bu-based MAC **(A)**. RIC showed a trend to decrease aGVHD, but it was not statistically significant **(B)**. RIC had the same cGVHD as MAC **(C)**. RIC, reduced intensity conditioning; MAC, myeloablative conditioning; TBI, total body irradiation; Bu, busulfan.

RIC significantly (*p* = 0.002) reduced NRM compared with TBI/Bu-based MAC (HR = 0.53, 95% CI 0.36–0.8) without heterogeneity (*p* = 0.40, *I* ^2^ = 0%) (**Figure 4A**). However, the treosulfan 30 g/m^2^-based MAC (8) significantly (*p* = 0.04) reduced NRM compared with RIC (HR = 1.67, 95% CI 1.02–2.72). RIC did not show significant difference compared with treosulfan 42 g/m^2^-based MAC (MC-FludT.14/L Trial I (7); HR = 0.76, 95% CI 0.45–1.30, *p* = 0.32).

In addition, RIC showed a trend to reduce aGVHD (**Figure 4B**) and III–IV aGVHD (**Supplement 4**) compared with TBI/Bu-based MAC (HR = 0.79, 95% CI 0.60–1.03, *p* = 0.08) (RR = 0.61, 95% CI 0.36–1.04, *p* = 0.07) and with no significant (*p* = 0.15 and *p* = 0.19) heterogeneity (*I* ^2^ = 43% and *I* ^2^ = 39%), respectively. Similarly, in the Beelen et al. (8) and MC-FludT.14/L Trial I (7) studies, RIC did not show a significant difference from treosulfan-based MAC (either 30 g/m^2^ or 42 g/m^2^).

We did not find a difference between RIC and MAC in cGVHD (**Figure 4C**) and extensive cGVHD (**Supplement 4**) (HR = 1.01, 95% CI 0.79–1.28, *p* = 0.96 and RR = 1.03, 95% CI 0.77–1.37, *p* = 0.84, respectively) with significant (*p* = 0.08 and *p* = 0.09) heterogeneity (*I* ^2^ = 49% and *I* ^2^ = 51%), respectively, and no difference between RIC and MAC in the subgroup analyses was observed.

RIC showed a trend of increasing GF (OR 2.19, 95% CI 0.96–5.03, *p* = 0.06) without heterogeneity (*p* = 0.34, *I* ^2^ = 12%). Moreover, GF incidence in the RIC and MAC arms was rare, 2.57% (18 events in 701 participants) and 1.16% (8 events in 690 participants), respectively. RIC did not show significant difference from MAC on overall organ toxicity and oral mucositis, with significant heterogeneity. Furthermore, RIC significantly (*p* = 0.04 and *p* = 0.01) reduced renal and urinary disorders (RR 0.61, 95% CI 0.39–0.97) and infection (RR 0.87, 95% CI 0.78–0.97) without heterogeneity (**Supplement 4**).

We did subgroup analysis based on diseases (AML or MDS) for OS and CIR; however, we still could not eliminate heterogeneity. The results of subgroup analyses did not show significant difference between RIC and MAC on OS and CIR in either AML or MDS subgroups (**Supplement 5**). We repeated the meta-analyses for the OS, CIR, and long-term OS with the fixed-effect model because of their significant heterogeneity, and the results did not change the overall conclusions of these endpoints (**Supplement 6**). We also removed one study at a time and then repeated the meta-analysis in the sensitivity analysis. The pooled HRs ranged from 0.84 to 1.05 for OS and from 1.02 to 1.26 for CIR. Results after removing any study (including Beelen et al. (8) and Scott et al. (12) studies) were overall stable. After we removed the Scott et al. (12) study, the heterogeneity of CIR disappeared (**Supplement 7**) and the results of CIR did not change. Eight CML patients were included in the Ringdén et al. (11) study. After removing it in the sensitivity analysis, we did not observe significant changes in OS, CIR, and NRM results (**Supplement 8**).

The quality of evidence for the OS, CIR, LFS, and cGVHD endpoints was moderate. The quality of the NRM and aGVHD endpoints was high (**Supplements 9, 10**).

## Discussion

Retrospective studies and their meta-analyses cannot balance the baseline characteristics of patients among different treatment arms. Most patients in the RIC arm in these studies were older or had higher comorbidity burden, which might underestimate the efficacy and safety of RIC. Half of all finished RCTs [Bornhäuser et al. (9), Scott et al. (12) and Kröger et al. (10)] did not enroll enough participants as the studies had planned which limited their power to demonstrate the difference between RIC and MAC. All the finished studies cannot provide reliable evidence to evaluate RIC for AML in CR and MDS, so we need higher level of evidence on this issue. Our meta-analysis included six high-quality RCTs with 1,413 participants and published and unpublished data, which limit the risk of publication bias. It was then more powerful and covered more patients than previous studies. To date, our study is the first comprehensive meta-analysis of RCTs that combined HR value to clarify the efficacy and safety of RIC vs. MAC and provides the highest current level of evidence for this matter.

The risk that RIC may increase CIR is the main concern for physicians to prescribe these conditioning regimens. A study of Scott et al. (12) demonstrated that RIC significantly increased relapse and prompted physicians to select MAC first for fit patients. However, when we combined data from all available RCTs, we failed to show differences in CIR between RIC and MAC. The heterogeneity was reported in the Scott et al. (12) study. After we removed it in the sensitivity analysis, we did not observe heterogeneity between the remaining five studies and the results did not change (**Supplement 7**). The relapse rate is affected by many factors, including the cytogenetic and molecular biologic characteristics of diseases, minimal residual disease (MRD) before HSCT, and immunosuppressant adjustment protocol, among others (30–33). It was unfeasible that all factors before transplantation were similar in every study; hence, the CIR was expected to be heterogeneous between studies. In a large observational analysis by the EBMT that included 2,974 middle-aged AML patients, relapse incidence was higher in intermediate- or high-risk patients but not in low-risk patients in the RIC group (32, 33). Most of our included studies did not examine MRD before HSCT to stratify participants, which might influence the results as patients who were MRD-positive would have higher CIR after RIC more than after MAC (34, 35). In the Scott et al. study, nearly two-thirds of the AML participants were found to have commonly mutated genes in AML, after using next-generation sequencing techniques, and in these patients, RIC significantly increased CIR compared with MAC, whereas in the remaining third of participants in whom these genes were not detected, RIC had the same CIR as MAC (36). In addition, all of the six included studies used the same GVHD prophylaxis in RIC and MAC, but the dose-adjustment protocol of immunosuppressant that was appropriate for MAC might have increased CIR for RIC. Therefore, it was possible that there was heterogeneity between the included studies. Moreover, three RCTs demonstrated that RIC did not increase CIR in the long-term follow-up data (11, 26, 28). As there were limited long-term data reported in all the included studies, we could not combine the long-term CIR. However, as most of the relapses after HSCT occur within 2 years (35), we conclude that RIC conditioning regimens do not increase CIR more than MAC for AML in CR and MDS.

A more intensive conditioning regimen causes more serious tissue damage, which may result in more severe aGVHD (36). Therefore, RIC is expected to not only decrease organ toxicity and tissue damage but also cause less aGVHD and NRM than TBI/Bu-based MAC. Our meta-analysis showed a trend for RIC to decrease aGVHD and III–IV aGVHD compared with TBI/Bu-based MAC, but it was not statistically significant. We are still in need of more high-quality studies to confirm whether there is a difference between RIC and MAC on aGVHD and III–IV aGVHD incidences. Our results indicated that there was no difference in cGVHD between RIC and MAC and confirmed the incidence of cGVHD was not related to conditioning intensity (37). In the retrospective studies, RIC reduced NRM (4–6) but RCTs failed to demonstrate the reduction. Our meta-analysis confirmed that RIC significantly reduced NRM compared with TBI/Bu-based MAC. There was no heterogeneity, and the quality of evidence was high (**Supplement 10**). RCTs represent relatively small sample size, especially some RCTs did not include enough participants as planned, which might not be powerful enough to demonstrate the difference. We included all the RCTs, which expanded the sample size and provided more powerful evidence to clarify the difference. In addition, the four included studies in the RIC vs. TBI/Bu-based MAC subgroup analysis involved relatively young and fit patients but not old patients, and in this subgroup analysis, RIC still caused less NRM. Consequently, RIC significantly reduces NRM more than TBI/Bu-based MAC for both young and old patients.

Moreover, our results showed that RIC significantly reduced some organ toxicity and infections compared with MAC, which indicated that RIC was more tolerable than MAC. On the other hand, our result did not show the difference on mucositis between RIC and MAC as generally expected. We observed that the heterogeneity of the meta-analysis was significant, so future studies are needed to clarify the issue. RIC had a trend to increase GF compared with MAC, but it was not significant. We showed only 18 GFs out of 701 patients and 8 GFs out of 690 patients reported in the RIC and MAC groups, respectively. The incidence of GF in the two groups was rare. According to the evidence available, we conclude that RIC causes marginal GF.

According to our results, RIC had the same OS as MAC, but heterogeneity was significant. In the HSCT procedure, the individualized prescriptions of different physicians will inevitably interfere with the results. Therefore, heterogeneity is common in clinical studies on HSCT, even when all the included studies are RCTs. In this regard, we used fixed-effect model to verify the results and did not find differences between RIC and MAC on OS (**Supplement 6**). In the study by Beelen et al. (8), treosulfan 30 g/m^2^-based MAC, which caused less NRM than RIC, was used. Despite the fact that it was included in the meta-analysis, RIC did not increase OS compared to MAC. Moreover, RIC was still not different than MAC in OS after we excluded it in the sensitivity analysis (**Supplement 11**). A report from the Acute Leukemia Working Party of the EBMT, retrospectively included 883 RIC compared with 1,041 MAC and demonstrated that RIC increased OS for ≥50-year patients than MAC and had the same OS for ≤50-year patients as MAC (38). A large sample retrospective study also showed that there was no significant difference in long-term survival between RIC and MAC (39). Both studies also showed that RIC did not increase relapse. Our meta-analysis could not divide participants according to age, but our results also showed that RIC at least did not decrease OS compared to MAC. The RIC vs. TBI/Bu-based MAC subgroup analysis included more young patients, but RIC also showed no difference from MAC on OS. Furthermore, our long-term follow-up OS data meta-analysis showed that RIC did not decrease long-term OS compared with TBI/Bu-based MAC. Consequently, we concluded that RIC did not increase CIR but decreased NRM compared with traditional MAC regimens. It at least did not increase aGVHD and had the same cGVHD as MAC; as a result, RIC did not decrease OS. Therefore, we confirm there is no difference between RIC and MAC in OS for AML in CR and MDS.

In the RIC vs. treosulfan 30 g/m^2^-based MAC subgroup analysis, treosulfan caused less NRM than RIC and increased OS (8). Treosulfan is a novel myeloablative agent with less toxicity than Bu (24) and treosulfan-based MAC was named reduced-toxicity conditioning regimen (24). The subgroup analysis confirmed that treosulfan was less toxic than Bu and suggested that treosulfan 30 g/m^2^-based MAC was better than Bu- or TBI-based RIC. It was a promising result and provided new myeloablative agents that were higher than the traditional Bu or TBI. However, only one RCT finished until recently and the RIC vs. treosulfan 42 g/m^2^-based MAC subgroup analysis did not show that treosulfan caused less NRM than RIC (7). Hence, we need more high-quality studies to confirm the result.

There are some limitations of our meta-analysis. Firstly, a relatively small number of clinical trials were included. Secondly, in OS, CIR, and LFS meta-analyses, there was significant heterogeneity between included studies. We tried to explore the heterogeneity with subgroup analysis based on conditioning regimens and diseases, but it could not be eliminated. We then suggest that the reason for the heterogeneity was the difference in treatment details available from the different transplantation centers and the inevitable patient heterogeneity between included studies. Thirdly, not all the included studies used blinding to personnel and patients. Allo-HSCT is a treatment with high NRM (40) and the treatment details should be individualized to every patient; therefore, blinding to patients and personnel could not be maintained. Fourthly, because we used data extracted from published reports but not individual patient data, we could not perform subgroup analysis based on diseases (AML in CR and MDS) and age. MDS patients may have less relapse than AML and young patients tolerate MAC better than old patients; thus, RIC may demonstrate better results in MDS patients and elderly patients. Despite these limitations, our meta-analysis is still reliable and can be used to guide physicians’ clinical decisions.

RIC had the same OS and CIR as MAC for AML in CR and MDS and significantly decreased NRM more than TBI/Bu-based MAC. Furthermore, RIC was more tolerable and comfortable and caused marginal GF. RIC is equally effective as MAC. Therefore, RIC is also a good choice of conditioning regimen before allo-HSCT for patients with AML in CR and MDS and not only an alternative treatment to MAC for unfit patients. On the other hand, more high-quality studies should continue to focus on the OS and LFS comparing RIC with MAC. MRD, disease (AML or MDS), cytogenetic and molecular biologic characteristics, and age should be considered as classification factors in future studies to identify the factors from which patients will derive more benefit from RIC. In addition, future studies should attempt to improve GVHD prophylaxis that would be more appropriate for RIC. We also need more studies to compare treosulfan-based MAC with RIC.

## Data Availability Statement

The original contributions presented in the study are included in the article/**Supplementary Material**. Further inquiries can be directed to the corresponding author.

## Author Contributions

YS conceived and designed the study, searched and selected trials for inclusion, assessed methodological quality of included trials, extracted data, performed the statistical analysis, and wrote the article. ZY searched trials, selected trials for inclusion, assessed methodological quality of included trials, and extracted data. JD wrote and revised the review. TW wrote and revised the manuscript. All authors contributed to the article and approved the submitted version.

## Conflict of Interest

The authors declare that the research was conducted in the absence of any commercial or financial relationships that could be construed as a potential conflict of interest.

## Publisher’s Note

All claims expressed in this article are solely those of the authors and do not necessarily represent those of their affiliated organizations, or those of the publisher, the editors and the reviewers. Any product that may be evaluated in this article, or claim that may be made by its manufacturer, is not guaranteed or endorsed by the publisher.

## Abbreviations

aGVHD, Acute graft versus host disease; Allo-HSCT, Allogeneic hematopoietic stem cell transplantation; AML, Acute myeloid leukemia; Bu, Busulfan; cGVHD, Chronic graft versus host disease; CI, Confidence interval; CIR, Cumulative incidence of relapse; CR, Complete remission; Cy, Cyclophosphamide; Flu, Fludarabine; GF, Graft failure; GVHD, Graft versus host disease; Gy, Gray; HR, Hazard ratio; HSCT, Hematopoietic stem cell transplantation; LFS, Leukemia-free survival; MAC, Myeloablative conditioning; MDS, Myelodysplastic syndrome; MRD, Minimal residual disease; NRM, Non-relapse mortality; OR, Odds ratio; OS, Overall survival; RCTs, Randomized controlled trials; RIC, Reduced intensity conditioning; RR, Risk ratio; TBI, Total body irradiation.
