# Supplementary material for: Reduced Intensity Conditioning Followed by Allogeneic Hematopoietic Stem Cell Transplantation Is a Good Choice for Acute Myeloid Leukemia and Myelodysplastic Syndrome: A Meta-Analysis of Randomized Controlled Trials
Source: Front Oncol. 2021 Oct 7;11:708727. doi: 10.3389/fonc.2021.708727 (PMC8529065; doi:10.3389/fonc.2021.708727)

Supplement 1

PRISMA 2009 Checklist

Section/topic	#	Checklist item	Reported on page #
**TITLE**			
Title	1	Identify the report as a systematic review, meta-analysis, or both.	1
**ABSTRACT**			
Structured summary	2	Provide a structured summary including, as applicable: background; objectives; data sources; study eligibility criteria, participants, and interventions; study appraisal and synthesis methods; results; limitations; conclusions and implications of key findings; systematic review registration number.	2
**INTRODUCTION**			
Rationale	3	Describe the rationale for the review in the context of what is already known.	2-3
Objectives	4	Provide an explicit statement of questions being addressed with reference to participants, interventions, comparisons, outcomes, and study design (PICOS).	3
**METHODS**			
Protocol and registration	5	Indicate if a review protocol exists, if and where it can be accessed (e.g., Web address), and, if available, provide registration information including registration number.	3
Eligibility criteria	6	Specify study characteristics (e.g., PICOS, length of follow-up) and report characteristics (e.g., years considered, language, publication status) used as criteria for eligibility, giving rationale.	3-4
Information sources	7	Describe all information sources (e.g., databases with dates of coverage, contact with study authors to identify additional studies) in the search and date last searched.	3-4
Search	8	Present full electronic search strategy for at least one database, including any limits used, such that it could be repeated.	Supplement 2
Study selection	9	State the process for selecting studies (i.e., screening, eligibility, included in systematic review, and, if applicable, included in the meta-analysis).	4
Data collection process	10	Describe method of data extraction from reports (e.g., piloted forms, independently, in duplicate) and any processes for obtaining and confirming data from investigators.	4
Data items	11	List and define all variables for which data were sought (e.g., PICOS, funding sources) and any assumptions and simplifications made.	3,4
Risk of bias in individual studies	12	Describe methods used for assessing risk of bias of individual studies (including specification of whether this was done at the study or outcome level), and how this information is to be used in any data synthesis.	Page 4, Table 2 and Supplement 3
Summary measures	13	State the principal summary measures (e.g., risk ratio, difference in means).	4

Synthesis of results	14	Describe the methods of handling data and combining results of studies, if done, including measures of consistency (e.g., I^2) for each meta-analysis.	4

Section/topic	#	Checklist item	Reported on page #
Risk of bias across studies	15	Specify any assessment of risk of bias that may affect the cumulative evidence (e.g., publication bias, selective reporting within studies).	Table 2
Additional analyses	16	Describe methods of additional analyses (e.g., sensitivity or subgroup analyses, meta-regression), if done, indicating which were pre-specified.	5
**RESULTS**			
Study selection	17	Give numbers of studies screened, assessed for eligibility, and included in the review, with reasons for exclusions at each stage, ideally with a flow diagram.	Pages 5 and Figure 1
Study characteristics	18	For each study, present characteristics for which data were extracted (e.g., study size, PICOS, follow-up period) and provide the citations.	Table 1 and Supplement 3
Risk of bias within studies	19	Present data on risk of bias of each study and, if available, any outcome level assessment (see item 12).	Table 2 and Supplement 3
Results of individual studies	20	For all outcomes considered (benefits or harms), present, for each study: (a) simple summary data for each intervention group (b) effect estimates and confidence intervals, ideally with a forest plot.	Pages 5-7, Figures 2-4 and Supplement 4
Synthesis of results	21	Present results of each meta-analysis done, including confidence intervals and measures of consistency.	Pages 5-7, Figures 2-4 and Supplement 4
Risk of bias across studies	22	Present results of any assessment of risk of bias across studies (see Item 15).	5
Additional analysis	23	Give results of additional analyses, if done (e.g., sensitivity or subgroup analyses, meta-regression [see Item 16]).	Page 7 and Supplements 5-8 and 11

PRISMA 2009 Checklist

**DISCUSSION**			
Summary of evidence	24	Summarize the main findings including the strength of evidence for each main outcome; consider their relevance to key groups (e.g., healthcare providers, users, and policy makers).	Pages 7-12 and Supplements 9 and 10
Limitations	25	Discuss limitations at study and outcome level (e.g., risk of bias), and at review-level (e.g., incomplete retrieval of identified research, reporting bias).	12
Conclusions	26	Provide a general interpretation of the results in the context of other evidence, and implications for future research.	12
**FUNDING**			
Funding	27	Describe sources of funding for the systematic review and other support (e.g., supply of data); role of funders for the systematic review.	14

From: Moher D, Liberati A, Tetzlaff J, Altman DG, The PRISMA Group (2009). Preferred Reporting Items for Systematic Reviews and Meta-Analyses: The PRISMA Statement. PLoS Med 6(6): e1000097. doi:10.1371/journal.pmed1000097

For more information, visit: **www.prisma-statement.org**.

Supplement 2. Searching strategy

Two reviewers (YS and ZY) independently searched PubMed, Embase, Cochrane Central Register of Controlled Trials, Web of Science, Google Scholar, and clinical trial registry websites (Clinical trials and WHO clinical trial registry) between Jan 1, 1980 and July 1, 2020.

Our searching strategy was composed of the following three terms.

1. The “stem cell transplantation” term: we used the following terms and free words to comprehensively search the related words, “stem cell” “cord blood” “bone marrow” “Hematopoietic Stem Cells” “Bone Marrow Transplantation” ”Hematopoietic Stem Cell Transplantation” ”Cord Blood Stem Cell Transplantation” “umbilic*” “hematopoiet*” “ transplant” and “ transplantation”.

2. The “reduced intensity conditioning” terms: we used the following free words to comprehensively search the related words, “’reduc* or low or lower or less or lessen or lesser or decreas*’ and ‘condition* or prepar* or pretreat* or precondition*’” or “fludarabine or Flu or melphalan or Mel or RIC”.

3. The “malignant myeloid diseases” terms: we used the following terms and free words to comprehensively search the related words, “acute myeloid leukemia” ”Leukemia, Myeloid, Acute” ”Myelodysplastic Syndromes” ”refractory anemia” ”’myeloid or myeloblast* or myelogenous’ and ‘malignan* or leukemia or neoplasm? or tumor? or tumour? or blast?’”.

To identify RCT studies, we used the Cochrane highly sensitive search filters for identifying randomized trials in Medline and Embase.

We also searched conference proceedings from the European Hematology Association, American Society of Hematology, International Bone Marrow Transplant Registry and European Group for Blood and Marrow Transplantation for relevant abstracts. Additionally, we hand-searched PubMed-related articles, reference lists of identified articles and contacted study authors, manufacturers and specialists for further information of unpublished trials.

Pubmed searching strategy

#1 (stem cell) or (stem cells)

#2 umbilic* or hematopoiet*

#3 #1 and #2

#4 (cord blood) or (bone marrow) or ‘Hematopoietic Stem Cells’[Mesh Terms] or #3

#5 transplant or transplantation

#6 #4 and #5

#7 ‘Bone Marrow transplantation’ or ‘Hematopoietic Stem Cell Transplantation’ or ‘Cord Blood Stem Cell Transplantation’ [Mesh Terms]

#8 #6 or #7

#9 reduc* or low or lower or less or lessen or lesser or decreas*

#10 condition* or prepar* or pretreat* or precondition*

#11 #9 and #10

#12 fludarabine or Flu or melphalan or Mel or RIC

#13 #11 or #12

#12 myeloid or myeloblast* or myelogenous

#13 malignan* or leukemia or neoplasm? or tumor? or tumour? or blast?

#14 #12 and #13

#15 (acute myeloid leukemia) or (refractoty anemia)

#16 myelodysplas* or MDS or AML or ‘Leukemia, Myeloid, Acute’[Mesh Terms] or ‘Myelodysplastic Syndromes’ [Mesh Terms]

#17 #14 or #15 or #16

#18 randomized controlled trial[Publication Type] OR controlled clinical trial[Publication Type] OR randomized[Title/Abstract] OR randomised[Title/Abstract] OR placebo[Title/Abstract] OR randomly [Title/Abstract] OR groups[Title/Abstract] OR trial[Title/Abstract] OR RCT

#19 #8 and #11 and #17 and #18

Supplement 3. Tables of characteristics and risk of bias assessment of included studies.

Table 1. **Charateristics of Beelen et al. study**[8]

Methods	randomised, controlled trial
Participants	**Inclusion:** Patients were eligible if they had acute myeloid leukaemia in first or consecutive complete haematological remission (blast counts <5% in bone marrow) or myelodysplastic syndrome (blast counts <20% in bone marrow) according to WHO 2008 and were indicated for allogeneic HSCT, but considered at increased risk for standard myeloablative conditioning based on age (⩾50 years), a HCT-CI score higher than 2, or both.23 Eligibility also included age between 18 and 70 years, Karnofsky index of 60% or higher, and availability of a human leucocyte antigen (HLA)-identical sibling (matched-related donor) or HLA-identical unrelated donor (matched-unrelated donor) identified by molecular typing of the HLA gene loci A, B, C, DRB1, and DQB1 (one antigen disparity [class I], one allele disparity [class II], or both were accepted).<br>**Exclusion:** substantial vital organ function impairment, previous allogeneic HSCT, and active and non-controlled infectious diseases under treatment, including active viral liver infection (see trial protocol in ClinicalTrials.gov).
Interventions	**RIC group**: The reduced-intensity conditioning regimen was 0·8 mg/kg busulfan applied as 2-h infusion at 6-h intervals on days –4 and –3 with 30 mg/m^2 fl udarabine daily for 4 days (day −6 to −3).<br>**MAC group**: Patients had treosulfan dose of 14 g/m^2 daily on days –6 to –4 of the 6-day regimen, was changed to 10 g/m^2 treosulfan daily applied as a 2-h infusion for 3 days (days –4 to –2) with 30 mg/m^2 fl udarabine daily for 4 days (day −6 to −3).<br>**GVHD prophylaxis:** Prophylaxis for GvHD was standardised in both groups and based on ciclosporin from day –1 (5 mg/kg daily, concentration adapted) and short course methotrexate (15 mg/m^2 on day +1, and 10 mg/m^2 on days +3 and +6). All matched unrelated donors recipients received anti-T-lymphocyte immune globulin (either ATG Fresenius or Grafalon Neovii at a dose of 10

	mg/kg on days –4, –3, and –2; or Thymoglobulin [Sanofi Genzyme] at a dose of 2·5 mg/kg on days –2 and –1).
Outcomes	**The primary efficacy outcome**: event-free survival 2 years after HSCT<br>**The secondary outcome**: overall survival, cumulative incidence of relapse or progression, cumulative incidence of non-relapse mortality (probability of dying without relapse or progression), and cumulative incidence of acute and chronic GvHD within 2 years of transplantation; incidence of grade 3–4 mucositis and other grade 3–4 adverse events between day –6 and day +28 after transplantation; cumulative incidence of engraftment on day +28; and incidence of complete donor-type chimerism on days +28 and +100 after transplantation

Table 2. Risk assessment of Beelen et al. study[8]

Bias	Authors' judgement	Support for judgement
Random sequence generation (selection bias)	Low risk	Quote: "A computergenerated and balanced (1:1) randomisation, using a permuted block technique with stratification by donor type"<br>Comment: Probably done.
Allocation concealment (selection bias)	Low risk	Quote: "Randomisation was centralised at the sponsor's clinical trial management site."<br>Comment: Probably done.
Blinding of participants and personnel (performance bias)<br>All outcomes	Low risk	Quote: "Study treatment was not blinded"<br>Comment: Probably not done.
Blinding of outcome assessment (detection bias)<br>All outcomes	Low risk	Quote: "the sponsor's staff including the biometrical group, investigators, and contracted research organisations were masked to aggregated analyses until the database lock for final confirmatory analysis."<br>Comment: Probably done.
Incomplete outcome data (attrition bias)<br>All outcomes	Low risk	Most of randomized participants had the primary outcome data reported<br>Comment: Probably done.
Selective reporting (reporting bias)	Low risk	The primary and secondary outcomes were all reported.<br>Comment: Probably done
Other bias	Unclear risk	Company organized

Table 3. Charateristics of Bornhäuser et al. study[9]

Methods	randomised, controlled trial
Participants	**Inclusion:** patients were aged 18–60 years and had AML in first complete remission. To be deemed in complete remission before transplantation, patients needed less than 5% blasts in the bone marrow and haematological recovery (>1000 neutrophils per μL and $>10^{11}$ platelets per L). Other inclusion criteria were intermediate-risk or high-risk AML defined by cytogenetics.<br>**Exclusion:** Patients with t(15;17) or t(8;21) AML were excluded from the trial.
Interventions	**RIC group**: The reduced-intensity conditioning regimen was four 2 Gy doses of total-body irradiation (8 Gy) on days −3 and −2 with 30 mg/m² fl udarabine daily for 4 days (day −6 to −3). Total-body irradiation could also be done on days −6 and −5 if logistics prevented treatment on days −3 and −2.<br>**MAC group**: Patients in the standard conditioning group had six 2 Gy doses of total-body irradiation (12 Gy) during 3 days (day −6 to −4; two doses per day) with lung shielding and 60 mg/kg cyclophosphamide per day for 2 days (days −3 and −2). A prophylactic infusion of mesna was given before and during cyclophosphamide treatment.<br>**GVHD prophylaxis:** 20 mg/kg of ATG-Fresenius (Fresenius Biotech, Bad Homburg, Germany) was given on days −3 to −1 (cumulative dose 60 mg/kg). Ciclosporin from day –1, with target trough concentrations of 200 ng/mL or higher, and 15 mg/m² methotrexate on day 1 and 10 mg/m² on days 3, 6, and 11.
Outcomes	**The primary efficacy outcome**: the incidence of nonrelapse mortality<br>**The secondary outcome**: overall survival, disease-free survival, relapse incidence, and incidence of acute and chronic graft-versus host disease.

Table 4. Risk assessment of Bornhäuser et al. study[9]

Bias	Authors' judgement	Support for judgement
Random sequence generation (selection bias)	Low risk	Quote: "patients were randomly assigned (1:1) via a computer-based ad-hoc minimisation procedure"<br>Comment: Probably done.

Allocation concealment (selection bias)	Low risk	Quote: “which was independent from all study sites, did the randomisation and allocated patients to treatment groups”<br>Comment: Probably done.
Blinding of participants and personnel (performance bias)<br>All outcomes	Low risk	Quote: “Study treatment was not blinded”<br>Comment: Probably not done.
Blinding of outcome assessment (detection bias)<br>All outcomes	Low risk	Quote: “Data were collected and certified centrally at the principal investigator’s site”<br>Comment: Probably done.
Incomplete outcome data (attrition bias)<br>All outcomes	Low risk	Most of randomized participants had the primary outcome data reported<br>Comment: Probably done.
Selective reporting (reporting bias)	Low risk	The primary and secondary outcomes were all reported.<br>Comment: Probably done
Other bias	Low risk	No

Table 5. Charateristics of Kröger et al. study[10]

Methods	randomised, controlled trial
Participants	**Inclusion:** cytologically proven MDS and sAML with , 20% of blasts at time of transplantation, a matched related or unrelated donor (one mismatch was allowed), age 18 to 60 years for unrelated donors and age 50 to 65 years for related donors, which was amended in 02/2006 to age 18 to 65 years. Other inclusion criteria were adequate hepatic, renal, pulmonary, and cardiac functions.
Interventions	**RIC group:** busulfan (8 mg/kg orally or 6.4 mg/kg intravenously) and fludarabine (150 mg/m2)<br>**MAC group:** busulfan (16 mg/kg orally or 12.8 mg/kg intravenously) and cyclophosphamide (120 mg/kg)<br>**GVHD prophylaxis:** cyclosporine and a short course of methotrexate (10 mg/m2 on days +1, +3, +6, and +11); In the case of unrelated donor antilymphocyte globulin (Fresenius, Graefelfing, Germany) at a cumulative does of 30 to 60 mg/kg or antithymocyte globulin (Thymoglobulin; Sanofi, Paris, France) at a cumulative dose of 6 to 10 mg/kg could be administered divided on days 23, 22, and 21 or alemtuzumab 100 mg divided on days 28 to 24 according to center policy; however,

	alemtuzumab was not used in a single patient.
Outcomes	**The primary efficacy outcome**: NRM after 1 year<br>**The secondary outcome**: comparison of engraftment, toxicity, acute and chronic GVHD, infectious complications, and event-free survival and overall survival at 2 years.

Table 6. Risk assessment of Kröger et al. study[10]

Bias	Authors' judgement	Support for judgement
Random sequence generation (selection bias)	Low risk	Quote: "Patients were randomly assigned in a 1:1 ratio and stratified according to donor (related v unrelated) blasts…"<br>Comment: Probably done.
Allocation concealment (selection bias)	Low risk	Quote: "Patients were randomly assigned in a 1:1 ratio and stratified according to donor (related v unrelated) blasts…"<br>Comment: Probably done.
Blinding of participants and personnel (performance bias)<br>All outcomes	Low risk	Open-label study.<br>Comment: Probably not done.
Blinding of outcome assessment (detection bias)<br>All outcomes	Low risk	Not mentioned<br>Comment: Probably not done.
Incomplete outcome data (attrition bias)<br>All outcomes	Low risk	Most of randomized participants had the primary outcome data reported<br>Comment: Probably done.
Selective reporting (reporting bias)	Low risk	The primary and secondary outcomes were all reported.<br>Comment: Probably done
Other bias	Low risk	No

Table 7. Characteristics of MC-FludT.14/L Trial I study[7]

Methods	randomised, controlled trial
Participants	**Inclusion**: 1. Patients with AML or MDS per WHO 2008 indicated for allogeneic HSCT but at increased risk for standard conditioning if aged ⩾ 50 years at transplant and/or had a HSCT -Comorbidity Index (HCT-CI) score ><br>2. Availability of a HLA-identical sibling donor (MRD) or HLA-identical unrelated donor (MUD).<br>3. Adult patients 18 to 70 years of age

	4. Karnofsky Index ≥ 60%<br>5. Men capable of reproduction and women of childbearing potential had to consent to using a highly effective method of birth control while on treatment and for at least 6 months thereafter.<br>**Exclusion**: The first criterion was different in France, based on the French competent authority's request:<br>Applied to Germany, Hungary, Italy, Poland:<br>1. Patients with acute promyelocytic leukaemia with t(15;17)(q22;q12) and in CR1.<br>Applied to France only:<br>• Patients with acute promyelocytic leukaemia with t(15;17)(q22;q12) and in CR1.<br>Patients with cytogenetic favourable AML ("low risk") and in CR1, who did not present unfavourable features like secondary or therapy-related AML or insufficient response to AML induction therapy.<br>MDS patients with IPSS-R "very low risk" or "low risk" at trial entry, who did not present unfavourable clinical features during disease history like refractory severe thrombocytopaenia with severe bleeding complications, life-threatening infectious complications due to severe neutropaenia and/or very high red blood cell transfusion requirement and related complications.
Interventions	**RIC group**: Busulfan 3.2 mg/kg/d (4 x 0.8 mg/kg) IV (2 hours infusion) on Day -4, -3. Fludarabine 30 mg/m²/d IV Day -6 to -2. All patients were to receive on Day 0 the allogeneic stem cell grafts from either a MRD or a MUD.<br>**MAC group**: Treosulfan 10 g/m² BSA IV (2 hours infusion) on Day -4, -3, -2. Fludarabine 30 mg/m²/d IV Day -6 to -2. (Treosulfan was given prior to fludarabine if both drugs were given on the same day).<br>**GVHD prophylaxis:** Ciclosporin 5 mg/kg/day PO Day -1 until day +100 (level adapted, treatment starts IV 3 mg/kg/day); Methotrexate (MTX) 15 mg/m² IV Day +1<br>10 mg/m² IV Day +3, +6, Ca-Folinate 15 mg/m² IV Day +1 (6 h after MTX) 10 mg/m² IV Day +3, +6; ATG for MUD only: ATG-S-Fresenius /Grafalon® 10 mg/kg IV Day -4, -3, -2 FR: ATG-Thymoglobuline 2.5 mg/kg IV Day -2, -1.
Outcomes	**The primary efficacy outcome**: Event-free survival (EFS) within 2 years after transplantation. Events were defined as relapse of disease, graft failure or death (whatever occurred first).

	**The secondary outcome**: Relapse/progression incidence (RI) within 2 years of HSCT, Overall survival (OS), Non-relapse mortality (NRM), Transplantation-related mortality (TRM).

Table 8. Risk assessment of MC-FludT.14/L Trial I study[7]

**Bias**	**Authors' judgement**	**Support for judgement**
Random sequence generation (selection bias)	Low risk	Quote: "Randomisation in a 1:1 ratio used software SAS Version 9.4 and a permuted block technique"<br>Comment: Probably done.
Allocation concealment (selection bias)	Low risk	Quote: "Randomisation in a 1:1 ratio used software SAS Version 9.4 and a permuted block technique"<br>Comment: Probably done.
Blinding of participants and personnel (performance bias)<br>All outcomes	Low risk	Quote: "No blinding was performed"<br>Comment Probably not done.
Blinding of outcome assessment (detection bias)<br>All outcomes	Low risk	Quote: "No blinding was performed".<br>Comment: Probably not done.
Incomplete outcome data (attrition bias)<br>All outcomes	Low risk	All randomized participants had the primary and secondary outcome data reported.<br>Comment: Probably done.
Selective reporting (reporting bias)	Low risk	The primary and secondary outcomes were all reported.<br>Comment: Probably done
Other bias	Unclear risk	Company organized.

Table 9. Characteristics of Ringdén et al. study[11]

Methods	Randomised, open-label non-inferiority trial
Participants	**Inclusion**: adult patients 60 years of age with acute myeloid leukaemia (AML) in first or second complete remission or with chronic myeloid leukaemia (CML) in chronic phase.<br>**Exclusion**: patients who would not be expected to tolerate MAC, had advanced disease, had HLA-mismatched grafts or had previously undergone transplantation
Interventions	**RIC group:** fludarabine 30 mg m^2 day^{-1} for 6 days, combined with busulfan 4 mg kg^{-1} day^{-1} for 2 days in patients with AML and in two patients with

	CML. Further two patients with CML received fludarabine 30 mg m^{-2} day^{-1} for 3 days combined with 2 Gy total body irradiation (TBI)<br>**MAC group:** busulfan 4 mg kg^{-1} day^{-1} administered in four doses for 4 days, combined with cyclophosphamide 120 mg kg^{-1}<br>**GVHD prophylaxis:** Most patients received cyclosporine (CsA) combined with four doses of methotrexate. CsA was administered intravenously (i.v.) on day 1 and on day 0, followed by oral CsA at a dose ranging between 3 and 12 mg kg^{-1}. We aimed to achieve a trough level of 100 ng mL^{-1} in HLA-identical sibling transplantation for malignancies. CsA trough target levels were between 200 and 300 ng mL^{-1} in patients with a nonmalignant disorder and a sibling donor, and in all patients with MUDs. In two patients who were given fludarabine and 2 Gy TBI, CsA was combined with mycophenolate mofetil. Two patients in the RIC group were treated with tacrolimus (0.1 mg kg^{-1} day^{-1} orally), aiming at trough levels between 5 and 15 ng mL^{-1}, in combination with sirolimus (3–6 mg) to achieve a trough level of 5–10 ng mL^{-1}. Anti-thymocyte globulin (Thymoglobulin; Genzyme, Cambridge, MA, USA) at a dose of 6–8 mg kg^{-1} was given to all recipients of unrelated grafts and to four patients conditioned with RIC.
Outcomes	leukaemia-free survival, overall survival, TRM, GVHD and relapse rate

Table 10. Risk assessment of Ringdén et al. study[11]

Bias	Authors' judgement	Support for judgement
Random sequence generation (selection bias)	Low risk	The authors used two envelopes and when the two were taken, two new envelopes were added to replace those that were used. We got the information by asking the authors through e-mail.<br>Comment: Probably done.
Allocation concealment (selection bias)	Low risk	The randomisation was performed by a research nurse not participating in the study or the care of the

		patients. We got the information by asking the authors through e-mail.<br>Comment: Probably done.
Blinding of participants and personnel (performance bias)<br>All outcomes	Low risk	Not mentioned.<br>Comment: Probably not done.
Blinding of outcome assessment (detection bias)<br>All outcomes	Low risk	Not mentioned.<br>Comment: Probably not done.
Incomplete outcome data (attrition bias)<br>All outcomes	Low risk	All randomized participants had the primary outcome data reported<br>Comment: Probably done.
Selective reporting (reporting bias)	Low risk	All outcomes were reported<br>Comment: Probably done
Other bias	Low risk	No

Table 11. Characteristics of Scott et al. study[12]

Methods	randomised, controlled trial
Participants	**Inclusion**: Participants had a WHO-defined diagnosis of AML or MDS, were undergoing a first HCT, and had＜5% marrow myeloblasts pre-HCT. Patients were 18 to 65 years of age and had an HLA-A, -B, and -DRB1 (6/6) –matched sibling donor or a ≥7/8 HLA-A, -B, -C, and -DRB1–matched unrelated donor and an HCT comorbidity index ≤ 4.<br>In AML, a composite definition of high risk included unfavorable risk cytogenetics according to the Eastern Cooperative Oncology Group/SWOG cytogenetic classification schema, presence of FLT3 mutation regardless of cytogenetic abnormalities, or three or more complete remissions. High-risk MDS was defined as patients with intermediate-II or high-risk disease per the International Prognostic Scoring System.
Interventions	**RIC group**: fludarabine (120 to 180 mg/m^2) with busulfan (≤ 8 mg/kg orally or 6.4 mg/kg intravenously; Flu/Bu2) or melphalan (≤ 150 mg/m2; Flu/Mel)<br>**MAC group**: busulfan (16 mg/kg orally or 12.8 mg/kg intravenously) with cyclophosphamide (120 mg/kg) or fludarabine (120 to 180 mg/m2; Flu/Bu4) or cyclophosphamide (120mg/kg) and total-body irradiation (12 to 14.2Gy)<br>**GVHD prophylaxis:** included methotrexate 10 to 15 mg/m2 on day 1 and 5 to 10 mg/m^2 on days 3, 6, and 11

	administered with cyclosporine or tacrolimus or tacrolimus with sirolimus or cyclosporine with mycophenolate mofetil. Experimental GVHD therapies were allowed provided they included a calcineurin inhibitor and no post-HCT cyclophosphamide or T-cell depletion. Antithymocyte globulin was allowed; however, its use was declared pre–random assignment, and it was administered regardless of conditioning intensity. Stem-cell sources were bone marrow or peripheralblood stem cells.
Outcomes	**The primary efficacy outcome**: OS at 18 months<br>**The secondary outcome**: relapse-free survival (RFS), TRM, absolute neutrophil count (ANC) and platelet recovery, kinetics of donor cell engraftment, graft failure, GVHD, grade 3 to 4 toxicities, infections, and quality of life

Table 12. Risk assessment of Scott et al. study[12]

**Bias**	**Authors' judgement**	**Support for judgement**
Random sequence generation (selection bias)	Low risk	Quote: "Patients were randomly assigned at a one-to-one ratio to either MAC and RIC using permuted blocks of random sizes with stratification by center"<br>Comment: Probably done.
Allocation concealment (selection bias)	Low risk	Quote: "Patients were randomly assigned at a one-to-one ratio to either MAC and RIC using permuted blocks of random sizes with stratification by center"<br>Comment: Probably done.
Blinding of participants and personnel (performance bias)<br>All outcomes	Low risk	Quote: "Patients and physicians were informed of the random assignment"<br>Comment Probably not done.
Blinding of outcome assessment (detection bias)<br>All outcomes	Low risk	Quote: "study investigators assigned to evaluate end points were blinded to each participant's random assignment.".<br>Comment: Probably done.
Incomplete outcome data (attrition bias)<br>All outcomes	Low risk	All randomized participants had the primary outcome data reported.<br>Comment: Probably done.
Selective reporting (reporting bias)	Low risk	The primary and secondary outcomes were all reported.<br>Comment: Probably done
Other bias	Low risk	No

Supplement 4

III-IV acute graft versus host disease

Study or Subgroup	Experimental Events	Experimental Total	Control Events	Control Total	Weight	Risk Ratio M-H, Fixed, 95% CI
**3.4.1 RIC vs. TBI/Bu based MAC**						
Kröger et al.	10	64	9	61	29.1%	1.06 [0.46, 2.43]
Ringdén et al.	0	18	4	19	13.8%	0.12 [0.01, 2.03]
Scott et al.	9	133	18	132	57.1%	0.50 [0.23, 1.06]
**Subtotal (95% CI)**		**215**		**212**	**100.0%**	**0.61 [0.36, 1.04]**
Total events	19		31			
Heterogeneity: Chi² = 3.28, df = 2 (P = 0.19); I² = 39%						
Test for overall effect: Z = 1.83 (P = 0.07)						
**3.4.2 RIC vs. treosulfan 30 g/m² based MAC**						
Beelen et al.	23	240	14	220	100.0%	1.51 [0.80, 2.85]
**Subtotal (95% CI)**		**240**		**220**	**100.0%**	**1.51 [0.80, 2.85]**
Total events	23		14			
Heterogeneity: Not applicable						
Test for overall effect: Z = 1.26 (P = 0.21)						
**3.4.4 RIC vs. treosulfan 42 g/m² based MAC**						
MC-FludT.14/L Trial I	14	152	24	168	100.0%	0.64 [0.35, 1.20]
**Subtotal (95% CI)**		**152**		**168**	**100.0%**	**0.64 [0.35, 1.20]**
Total events	14		24			
Heterogeneity: Not applicable						
Test for overall effect: Z = 1.38 (P = 0.17)						
Test for subgroup differences: Chi² = 5.25, df = 2 (P = 0.07), I² = 61.9%						

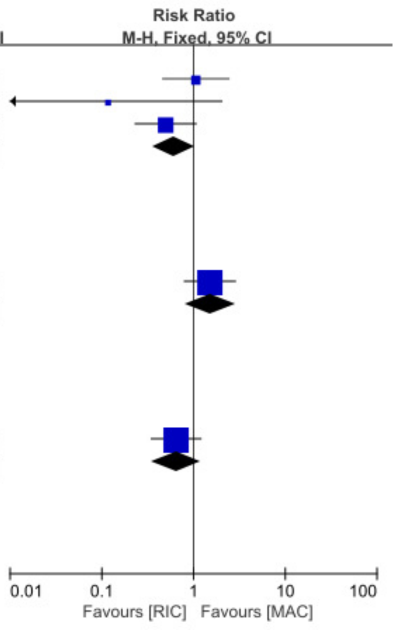

Extensive chronic graft versus host disease

Study or Subgroup	Experimental Events	Experimental Total	Control Events	Control Total	Weight	Risk Ratio M-H, Random, 95% CI
**3.5.1 RIC vs. TBI/Bu based MAC**						
Bornhäuser et al.	22	94	17	90	15.8%	1.24 [0.71, 2.18]
Kröger et al.	25	64	21	61	19.6%	1.13 [0.71, 1.80]
Scott et al.	29	133	45	132	22.5%	0.64 [0.43, 0.95]
**Subtotal (95% CI)**		**291**		**283**	**57.9%**	**0.94 [0.61, 1.44]**
Total events	76		83			
Heterogeneity: Tau² = 0.09; Chi² = 5.01, df = 2 (P = 0.08); I² = 60%						
Test for overall effect: Z = 0.29 (P = 0.77)						
**3.5.2 RIC vs. treosulfan 30 g/m² based MAC**						
Beelen et al.	42	190	28	179	21.0%	1.41 [0.92, 2.18]
**Subtotal (95% CI)**		**190**		**179**	**21.0%**	**1.41 [0.92, 2.18]**
Total events	42		28			
Heterogeneity: Not applicable						
Test for overall effect: Z = 1.57 (P = 0.12)						
**3.5.5 RIC vs. treosulfan 42 g/m² based MAC**						
MC-FludT.14/L Trial I	31	128	32	132	21.1%	1.00 [0.65, 1.54]
**Subtotal (95% CI)**		**128**		**132**	**21.1%**	**1.00 [0.65, 1.54]**
Total events	31		32			
Heterogeneity: Not applicable						
Test for overall effect: Z = 0.00 (P = 1.00)						
**Total (95% CI)**		**609**		**594**	**100.0%**	**1.03 [0.77, 1.37]**
Total events	149		143			
Heterogeneity: Tau² = 0.05; Chi² = 8.09, df = 4 (P = 0.09); I² = 51%						
Test for overall effect: Z = 0.21 (P = 0.84)						
Test for subgroup differences: Chi² = 2.01, df = 2 (P = 0.37), I² = 0.4%						

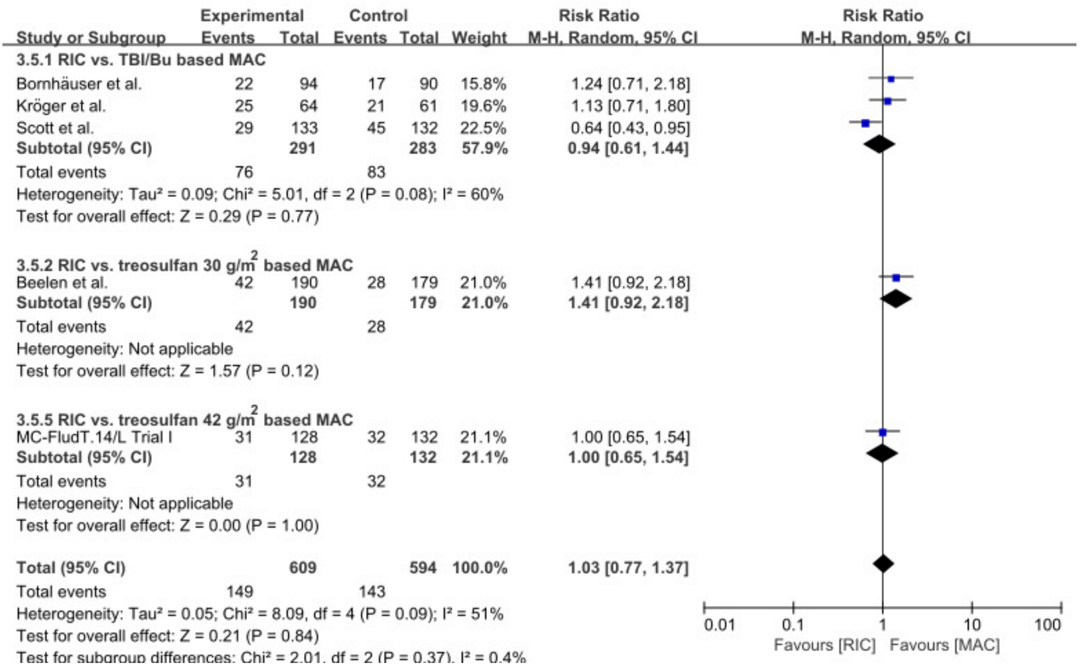

Graft failure

Study or Subgroup	RIC Events	RIC Total	MAC Events	MAC Total	Weight	Odds Ratio M-H, Fixed, 95% CI
**3.6.1 RIC vs. TBI/Bu based MAC**						
Bornhäuser et al.	0	94	0	90		Not estimable
Kröger et al.	3	64	4	61	48.0%	0.70 [0.15, 3.27]
Ringdén et al.	2	18	1	19	10.6%	2.25 [0.19, 27.22]
Scott et al.	3	133	1	132	12.1%	3.02 [0.31, 29.44]
**Subtotal (95% CI)**		**309**		**302**	**70.7%**	**1.33 [0.45, 3.92]**
Total events	8		6			
Heterogeneity: Chi² = 1.34, df = 2 (P = 0.51); I² = 0%						
Test for overall effect: Z = 0.52 (P = 0.61)						
**3.6.2 RIC vs. treosulfan 30 g /m² based MAC**						
Beelen et al.	8	240	0	220	6.2%	16.12 [0.93, 280.99]
**Subtotal (95% CI)**		**240**		**220**	**6.2%**	**16.12 [0.93, 280.99]**
Total events	8		0			
Heterogeneity: Not applicable						
Test for overall effect: Z = 1.91 (P = 0.06)						
**3.6.8 RIC vs. treosulfan 42 g /m² based MAC**						
MC-FludT.14/L Trial I	2	152	2	168	23.1%	1.11 [0.15, 7.95]
**Subtotal (95% CI)**		**152**		**168**	**23.1%**	**1.11 [0.15, 7.95]**
Total events	2		2			
Heterogeneity: Not applicable						
Test for overall effect: Z = 0.10 (P = 0.92)						
**Total (95% CI)**		**701**		**690**	**100.0%**	**2.19 [0.96, 5.03]**
Total events	18		8			
Heterogeneity: Chi² = 4.52, df = 4 (P = 0.34); I² = 12%						
Test for overall effect: Z = 1.86 (P = 0.06)						
Test for subgroup differences: Chi² = 2.75, df = 2 (P = 0.25), I² = 27.4%						

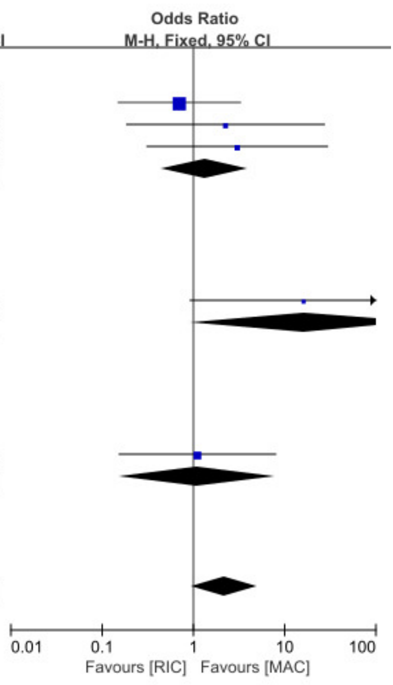

Overall organ toxicity and oral mucositis

Study or Subgroup	RIC Events	RIC Total	MAC Events	MAC Total	Weight	Risk Ratio M-H, Random, 95% CI
**4.1.1 Organ toxicity**						
Beelen et al.	131	240	118	221	30.5%	1.02 [0.86, 1.21]
Kröger et al.	31	64	39	61	18.4%	0.76 [0.55, 1.04]
MC-FludT.14/L Trial I	61	152	67	168	21.8%	1.01 [0.77, 1.32]
Scott et al.	74	133	98	132	29.3%	0.75 [0.62, 0.90]
**Subtotal (95% CI)**		**589**		**582**	**100.0%**	**0.88 [0.73, 1.06]**
Total events	297		322			
Heterogeneity: Tau² = 0.02; Chi² = 7.93, df = 3 (P = 0.05); I² = 62%						
Test for overall effect: Z = 1.38 (P = 0.17)						
**4.1.5 Oral mucositis**						
Beelen et al.	18	240	10	220	13.5%	1.65 [0.78, 3.50]
Bornhäuser et al.	77	93	85	88	19.8%	0.86 [0.78, 0.95]
Kröger et al.	23	64	34	61	17.6%	0.64 [0.43, 0.96]
MC-FludT.14/L Trial I	26	152	18	168	15.7%	1.60 [0.91, 2.79]
Ringdén et al.	8	18	15	19	15.7%	0.56 [0.32, 0.99]
Scott et al.	25	133	85	133	17.8%	0.29 [0.20, 0.43]
**Subtotal (95% CI)**		**700**		**689**	**100.0%**	**0.76 [0.47, 1.23]**
Total events	177		247			
Heterogeneity: Tau² = 0.30; Chi² = 49.72, df = 5 (P < 0.00001); I² = 90%						
Test for overall effect: Z = 1.11 (P = 0.27)						
Test for subgroup differences: Chi² = 0.31, df = 1 (P = 0.58), I² = 0%						

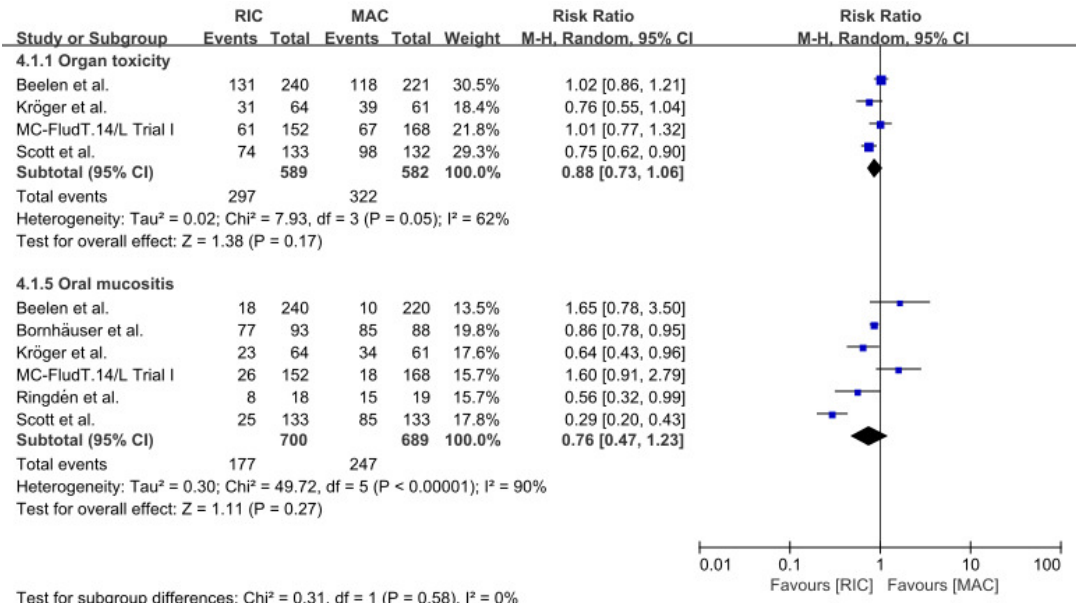

Specific organ toxicities and infection

Study or Subgroup	RIC Events	RIC Total	MAC Events	MAC Total	Weight	Risk Ratio M-H, Fixed, 95% CI
**4.2.2 Hepatobiliary disorders**						
Beelen et al.	2	240	1	221	2.2%	1.84 [0.17, 20.17]
Bornhäuser et al.	24	94	31	88	69.2%	0.72 [0.46, 1.13]
Kröger et al.	7	64	11	61	24.3%	0.61 [0.25, 1.46]
MC-FludT.14/L Trial I	2	152	1	168	2.1%	2.21 [0.20, 24.13]
Scott et al.	1	133	1	132	2.2%	0.99 [0.06, 15.70]
**Subtotal (95% CI)**		**683**		**670**	**100.0%**	**0.76 [0.52, 1.11]**
Total events	36		45			
Heterogeneity: Chi² = 1.62, df = 4 (P = 0.81); I² = 0%						
Test for overall effect: Z = 1.42 (P = 0.16)						
**4.2.3 Renal and urinary disorders**						
Beelen et al.	1	240	3	221	7.7%	0.31 [0.03, 2.93]
Bornhäuser et al.	17	94	24	88	61.2%	0.66 [0.38, 1.15]
Kröger et al.	4	64	9	61	22.8%	0.42 [0.14, 1.30]
MC-FludT.14/L Trial I	2	152	3	168	7.0%	0.74 [0.12, 4.35]
Scott et al.	1	133	0	132	1.2%	2.98 [0.12, 72.44]
**Subtotal (95% CI)**		**683**		**670**	**100.0%**	**0.61 [0.39, 0.97]**
Total events	25		39			
Heterogeneity: Chi² = 1.84, df = 4 (P = 0.77); I² = 0%						
Test for overall effect: Z = 2.09 (P = 0.04)						
**4.2.4 Cardiac disorders**						
Beelen et al.	7	240	7	220	35.4%	0.92 [0.33, 2.57]
Kröger et al.	1	64	4	61	19.8%	0.24 [0.03, 2.07]
MC-FludT.14/L Trial I	0	152	5	168	25.3%	0.10 [0.01, 1.80]
Ringdén et al.	0	18	0	19		Not estimable
Scott et al.	3	133	4	132	19.5%	0.74 [0.17, 3.26]
**Subtotal (95% CI)**		**607**		**600**	**100.0%**	**0.54 [0.26, 1.11]**
Total events	11		20			
Heterogeneity: Chi² = 3.04, df = 3 (P = 0.39); I² = 1%						
Test for overall effect: Z = 1.67 (P = 0.10)						
**4.2.6 Infection**						
Beelen et al.	58	240	58	220	19.5%	0.92 [0.67, 1.26]
Kröger et al.	44	64	48	61	15.8%	0.87 [0.71, 1.08]
MC-FludT.14/L Trial I	75	152	107	168	32.7%	0.77 [0.64, 0.94]
Ringdén et al.	3	18	5	19	1.6%	0.63 [0.18, 2.27]
Scott et al.	90	133	94	132	30.4%	0.95 [0.81, 1.12]
**Subtotal (95% CI)**		**607**		**600**	**100.0%**	**0.87 [0.78, 0.97]**
Total events	270		312			
Heterogeneity: Chi² = 2.85, df = 4 (P = 0.58); I² = 0%						
Test for overall effect: Z = 2.54 (P = 0.01)						
Test for subgroup differences: Chi² = 3.88, df = 3 (P = 0.28), I² = 22.6%						

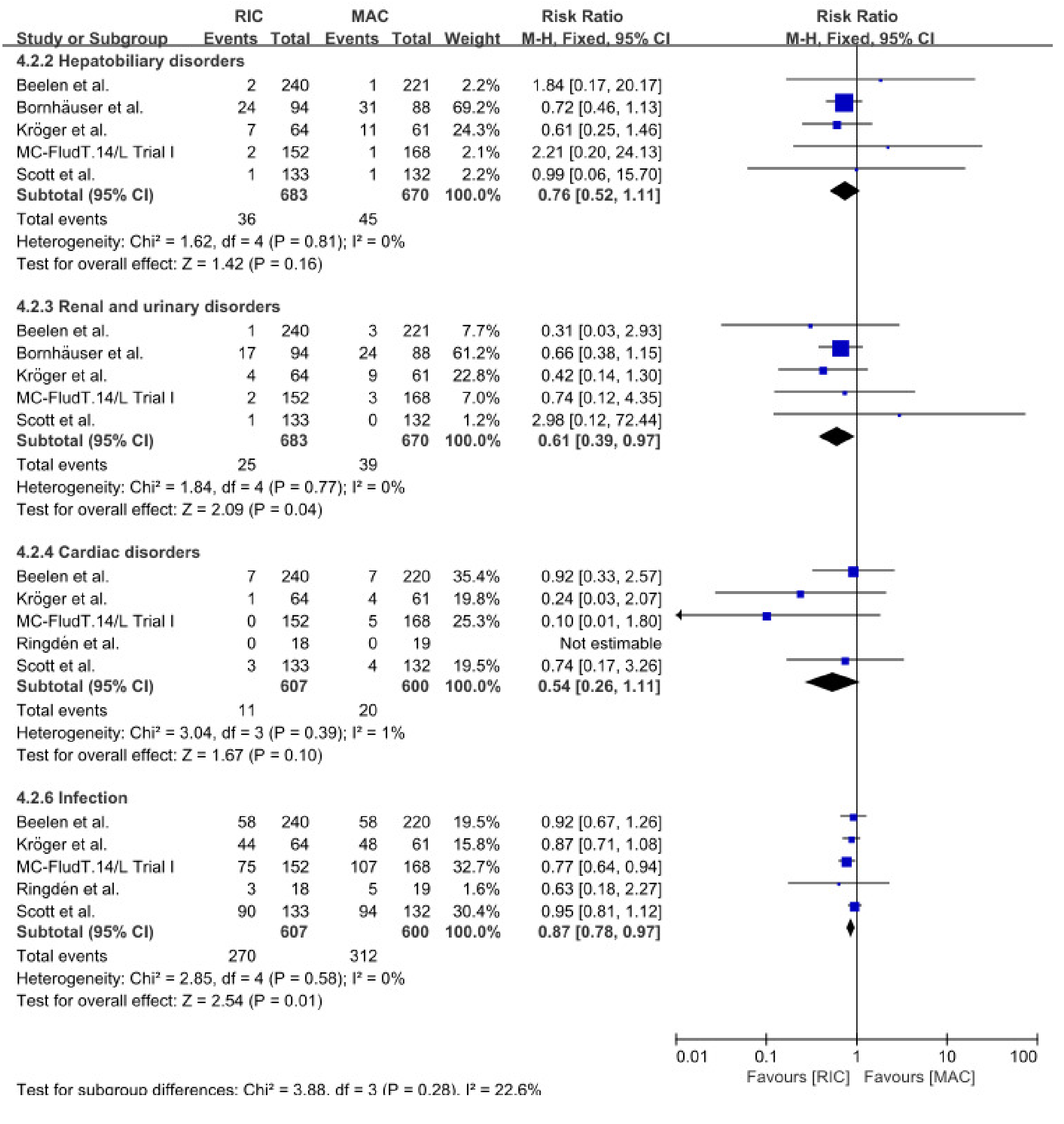

Supplement 5

OS endpoint subgroup analysis based on diseases

Study or Subgroup	log[Hazard Ratio]	SE	RIC Total	MAC Total	Weight	Hazard Ratio IV, Random, 95% CI
**2.6.1 AML**						
Ringdén et al.	-0.63	0.6	18	19	11.0%	0.53 [0.16, 1.73]
Bornhäuser et al.	-0.26	0.24	99	96	25.6%	0.77 [0.48, 1.23]
Scott et al.	0.54	0.24	110	108	25.6%	1.72 [1.07, 2.75]
**Subtotal (95% CI)**			**227**	**223**	**62.2%**	**0.99 [0.50, 1.94]**
Heterogeneity: Tau² = 0.24; Chi² = 7.08, df = 2 (P = 0.03); I² = 72%						
Test for overall effect: Z = 0.04 (P = 0.97)						
**2.6.2 MDS**						
Kröger et al.	-0.57	0.32	65	64	21.4%	0.57 [0.30, 1.06]
Scott et al.	-0.27	0.43	27	27	16.4%	0.76 [0.33, 1.77]
**Subtotal (95% CI)**			**92**	**91**	**37.8%**	**0.63 [0.38, 1.04]**
Heterogeneity: Tau² = 0.00; Chi² = 0.31, df = 1 (P = 0.58); I² = 0%						
Test for overall effect: Z = 1.80 (P = 0.07)						
**Total (95% CI)**			**319**	**314**	**100.0%**	**0.85 [0.53, 1.36]**
Heterogeneity: Tau² = 0.17; Chi² = 10.61, df = 4 (P = 0.03); I² = 62%						
Test for overall effect: Z = 0.68 (P = 0.50)						
Test for subgroup differences: Chi² = 1.10, df = 1 (P = 0.30), I² = 8.8%						

Hazard Ratio
IV, Random, 95% CI

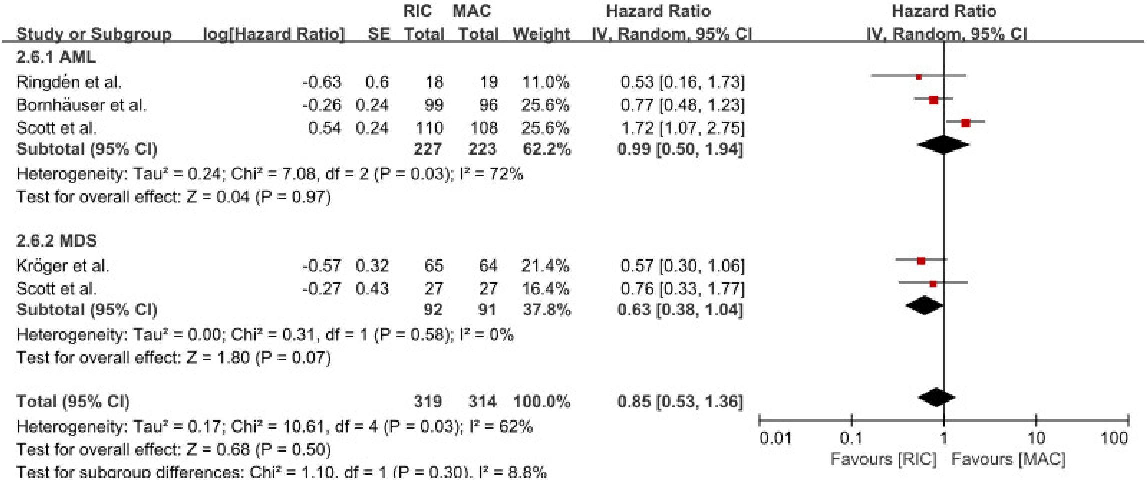

CIR endpoint subgroup analysis based on diseases

Study or Subgroup	log[Hazard Ratio]	SE	RIC Total	MAC Total	Weight	Hazard Ratio IV, Random, 95% CI
**2.7.1 AML**						
Bornhäuser et al.	-0.26	0.24	99	96	21.8%	0.77 [0.48, 1.23]
Ringdén et al.	-0.63	0.6	18	19	15.8%	0.53 [0.16, 1.73]
Scott et al.	0.76	0.22	110	108	22.1%	2.14 [1.39, 3.29]
**Subtotal (95% CI)**			**227**	**223**	**59.7%**	**1.05 [0.45, 2.45]**
Heterogeneity: Tau² = 0.44; Chi² = 12.03, df = 2 (P = 0.002); I² = 83%						
Test for overall effect: Z = 0.11 (P = 0.92)						
**2.7.2 MDS**						
Kröger et al.	-0.57	0.32	65	64	20.6%	0.57 [0.30, 1.06]
Scott et al.	1.8	0.38	27	27	19.7%	6.05 [2.87, 12.74]
**Subtotal (95% CI)**			**92**	**91**	**40.3%**	**1.83 [0.18, 18.70]**
Heterogeneity: Tau² = 2.69; Chi² = 22.76, df = 1 (P < 0.00001); I² = 96%						
Test for overall effect: Z = 0.51 (P = 0.61)						
**Total (95% CI)**			**319**	**314**	**100.0%**	**1.28 [0.57, 2.90]**
Heterogeneity: Tau² = 0.74; Chi² = 35.18, df = 4 (P < 0.00001); I² = 89%						
Test for overall effect: Z = 0.59 (P = 0.55)						
Test for subgroup differences: Chi² = 0.20, df = 1 (P = 0.66), I² = 0%						

Hazard Ratio
IV, Random, 95% CI

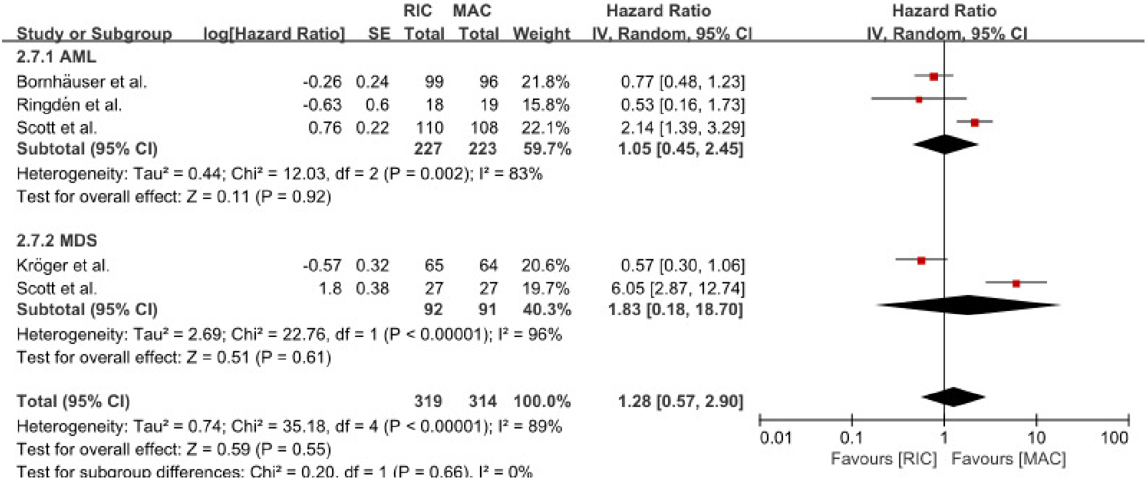

Supplement 6

Overall survival (OS) with fixed effect model

Study or Subgroup	log[Hazard Ratio]	SE	RIC Total	MAC Total	Weight	Hazard Ratio IV, Fixed, 95% CI
**2.1.1 RIC vs. TBI/Bu based MAC**						
Bornhäuser et al.	-0.26	0.24	99	96	16.4%	0.77 [0.48, 1.23]
Kröger et al.	-0.57	0.32	65	64	9.2%	0.57 [0.30, 1.06]
Ringdén et al.	-0.63	0.6	18	19	2.6%	0.53 [0.16, 1.73]
Scott et al.	0.42	0.23	137	135	17.8%	1.52 [0.97, 2.39]
**Subtotal (95% CI)**			**319**	**314**	**46.0%**	**0.92 [0.70, 1.22]**
Heterogeneity: Chi² = 8.47, df = 3 (P = 0.04); I² = 65%						
Test for overall effect: Z = 0.56 (P = 0.58)						
**2.1.2 RIC vs. treosulfan 30 g /m² based MAC**						
Beelen et al.	0.49	0.17	240	220	32.6%	1.63 [1.17, 2.28]
**Subtotal (95% CI)**			**240**	**220**	**32.6%**	**1.63 [1.17, 2.28]**
Heterogeneity: Not applicable						
Test for overall effect: Z = 2.88 (P = 0.004)						
**2.1.3 RIC vs. treosulfan 42 g /m² based MAC**						
MC-FludT.14/L Trial I	-0.26	0.21	152	168	21.4%	0.77 [0.51, 1.16]
**Subtotal (95% CI)**			**152**	**168**	**21.4%**	**0.77 [0.51, 1.16]**
Heterogeneity: Not applicable						
Test for overall effect: Z = 1.24 (P = 0.22)						
**Total (95% CI)**			**711**	**702**	**100.0%**	**1.07 [0.88, 1.29]**
Heterogeneity: Chi² = 18.14, df = 5 (P = 0.003); I² = 72%						
Test for overall effect: Z = 0.70 (P = 0.49)						
Test for subgroup differences: Chi² = 9.67, df = 2 (P = 0.008), I² = 79.3%						

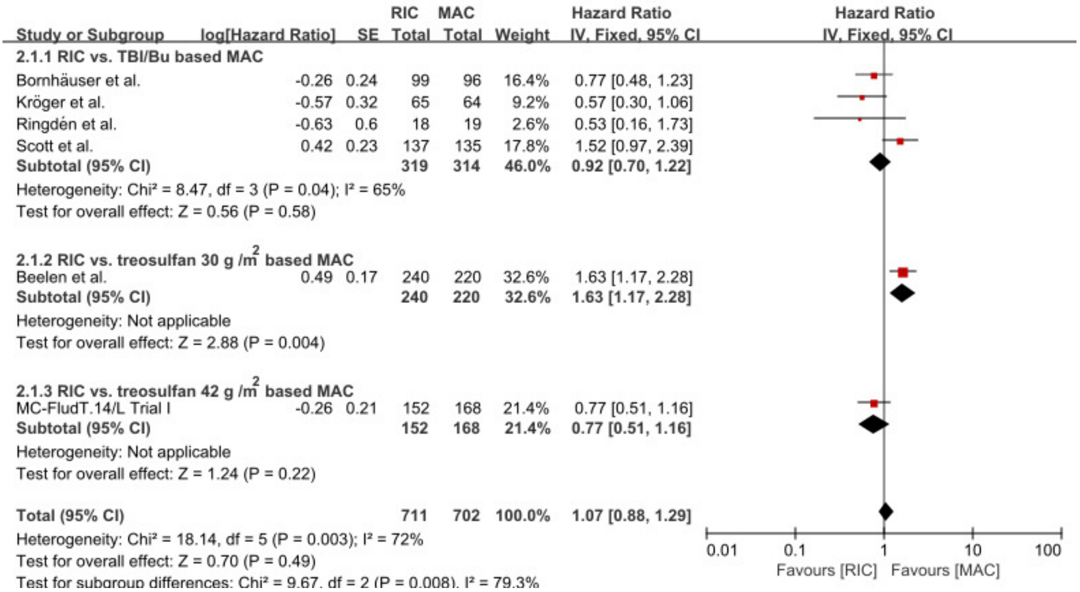

Cumulative incidence of relapse with fixed effect model

Study or Subgroup	log[Hazard Ratio]	SE	RIC Total	MAC Total	Weight	Hazard Ratio IV, Fixed, 95% CI
**2.2.1 RIC vs. TBI/Bu based MAC**						
Bornhäuser et al.	0.1	0.28	99	96	6.8%	1.11 [0.64, 1.91]
Kröger et al.	0.21	0.45	65	64	2.6%	1.23 [0.51, 2.98]
Ringdén et al.	-0.97	0.71	18	19	1.1%	0.38 [0.09, 1.52]
Scott et al.	0.72	0.22	137	135	10.9%	2.05 [1.33, 3.16]
**Subtotal (95% CI)**			**319**	**314**	**21.4%**	**1.46 [1.07, 1.99]**
Heterogeneity: Chi² = 7.15, df = 3 (P = 0.07); I² = 58%						
Test for overall effect: Z = 2.40 (P = 0.02)						
**2.2.2 RIC vs. treosulfan 30 g /m² based MAC**						
Beelen et al.	0.14	0.2	240	220	13.2%	1.15 [0.78, 1.70]
**Subtotal (95% CI)**			**240**	**220**	**13.2%**	**1.15 [0.78, 1.70]**
Heterogeneity: Not applicable						
Test for overall effect: Z = 0.70 (P = 0.48)						
**2.2.3 RIC vs. treosulfan 42 g /m² based MAC**						
MC-FludT.14/L Trial I	-0.01	0.09	152	168	65.4%	0.99 [0.83, 1.18]
**Subtotal (95% CI)**			**152**	**168**	**65.4%**	**0.99 [0.83, 1.18]**
Heterogeneity: Not applicable						
Test for overall effect: Z = 0.11 (P = 0.91)						
**Total (95% CI)**			**711**	**702**	**100.0%**	**1.10 [0.95, 1.27]**
Heterogeneity: Chi² = 11.80, df = 5 (P = 0.04); I² = 58%						
Test for overall effect: Z = 1.28 (P = 0.20)						
Test for subgroup differences: Chi² = 4.65, df = 2 (P = 0.10), I² = 57.0%						

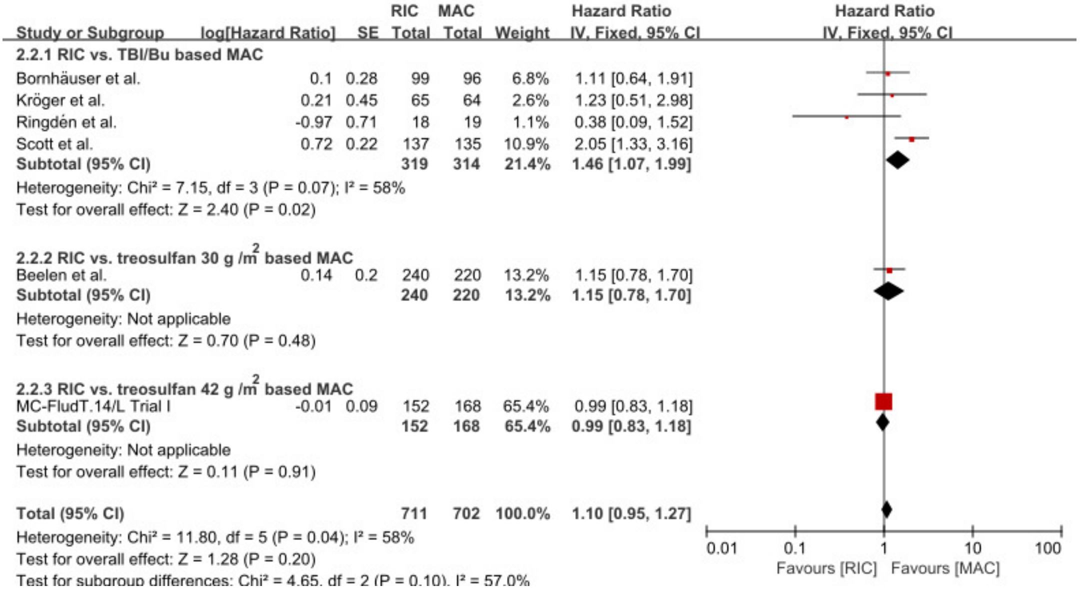

Long-term OS with fixed effect model

Study or Subgroup	log[Hazard Ratio]	SE	RIC Total	MAC Total	Weight	Hazard Ratio IV, Fixed, 95% CI
Bornhäuser et al.	-0.34	0.2	99	96	35.6%	0.71 [0.48, 1.05]
Kröger et al.	-0.38	0.26	65	64	21.1%	0.68 [0.41, 1.14]
Ringdén et al.	-0.63	0.6	18	19	4.0%	0.53 [0.16, 1.73]
Scott et al.	0.43	0.19	137	135	39.4%	1.54 [1.06, 2.23]
**Total (95% CI)**			**319**	**314**	**100.0%**	**0.95 [0.75, 1.19]**
Heterogeneity: Chi² = 11.03, df = 3 (P = 0.01); I² = 73%						
Test for overall effect: Z = 0.47 (P = 0.64)						

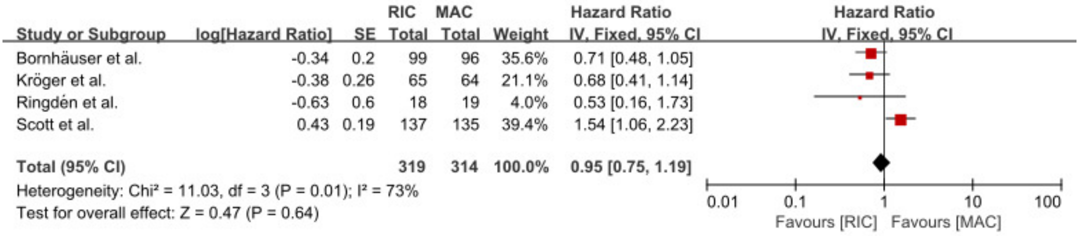

Supplement7

Cumulative incidence of relapse without Scott et al.

Study or Subgroup	log[Hazard Ratio]	SE	RIC Total	MAC Total	Weight	Hazard Ratio IV, Fixed, 95% CI
**2.2.1 RIC vs. TBI/Bu based MAC**						
Bornhäuser et al.	0.1	0.28	99	96	7.6%	1.11 [0.64, 1.91]
Kröger et al.	0.21	0.45	65	64	2.9%	1.23 [0.51, 2.98]
Ringdén et al.	-0.97	0.71	18	19	1.2%	0.38 [0.09, 1.52]
Scott et al.	0.72	0.22	137	135	0.0%	2.05 [1.33, 3.16]
**Subtotal (95% CI)**			**182**	**179**	**11.7%**	**1.02 [0.66, 1.59]**
Heterogeneity: Chi² = 2.20, df = 2 (P = 0.33); I² = 9%						
Test for overall effect: Z = 0.09 (P = 0.93)						
**2.2.2 RIC vs. treosulfan 30 g /m² based MAC**						
Beelen et al.	0.14	0.2	240	220	14.9%	1.15 [0.78, 1.70]
**Subtotal (95% CI)**			**240**	**220**	**14.9%**	**1.15 [0.78, 1.70]**
Heterogeneity: Not applicable						
Test for overall effect: Z = 0.70 (P = 0.48)						
**2.2.3 RIC vs. treosulfan 42 g /m² based MAC**						
MC-FludT.14/L Trial I	-0.01	0.09	152	168	73.4%	0.99 [0.83, 1.18]
**Subtotal (95% CI)**			**152**	**168**	**73.4%**	**0.99 [0.83, 1.18]**
Heterogeneity: Not applicable						
Test for overall effect: Z = 0.11 (P = 0.91)						
**Total (95% CI)**			**574**	**567**	**100.0%**	**1.02 [0.87, 1.18]**
Heterogeneity: Chi² = 2.67, df = 4 (P = 0.61); I² = 0%						
Test for overall effect: Z = 0.20 (P = 0.84)						
Test for subgroup differences: Chi² = 0.47, df = 2 (P = 0.79), I² = 0%						

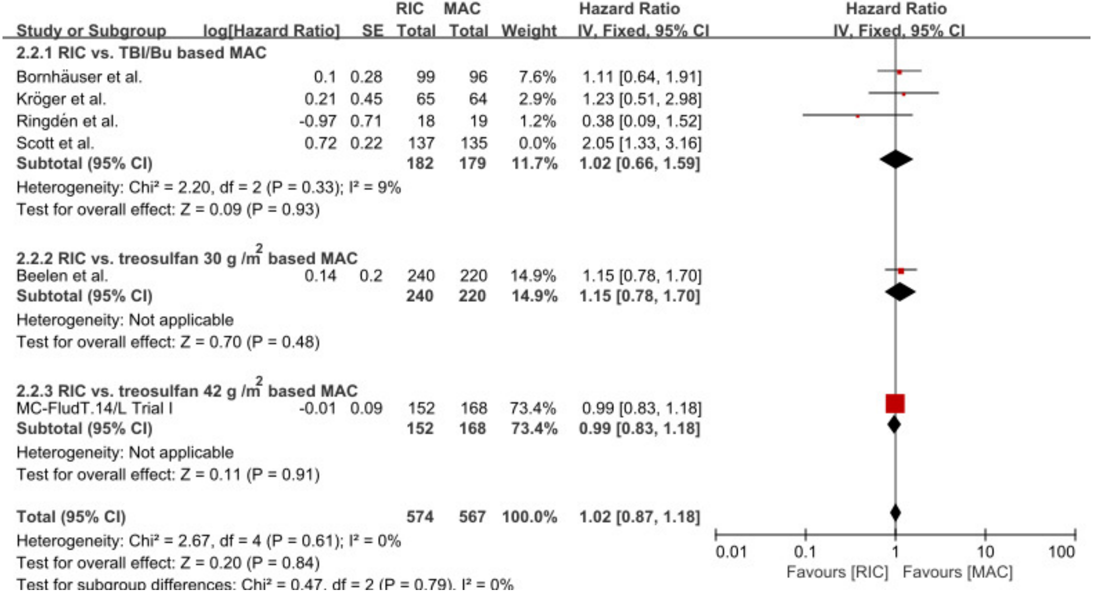

Supplement 8

Overall survival without Ringdén et al.

Study or Subgroup	log[Hazard Ratio]	SE	RIC Total	MAC Total	Weight	Hazard Ratio IV, Random, 95% CI
**2.1.1 RIC vs. TBI/Bu based MAC**						
Bornhäuser et al.	-0.26	0.24	99	96	19.7%	0.77 [0.48, 1.23]
Kröger et al.	-0.57	0.32	65	64	16.4%	0.57 [0.30, 1.06]
Ringdén et al.	-0.63	0.6	18	19	0.0%	0.53 [0.16, 1.73]
Scott et al.	0.42	0.23	137	135	20.2%	1.52 [0.97, 2.39]
**Subtotal (95% CI)**			**301**	**295**	**56.3%**	**0.90 [0.50, 1.59]**
Heterogeneity: Tau^2 = 0.19; Chi^2 = 7.58, df = 2 (P = 0.02); I^2 = 74%						
Test for overall effect: Z = 0.37 (P = 0.71)						
**2.1.2 RIC vs. treosulfan 30 g /m^2 based MAC**						
Beelen et al.	0.49	0.17	240	220	22.7%	1.63 [1.17, 2.28]
**Subtotal (95% CI)**			**240**	**220**	**22.7%**	**1.63 [1.17, 2.28]**
Heterogeneity: Not applicable						
Test for overall effect: Z = 2.88 (P = 0.004)						
**2.1.3 RIC vs. treosulfan 42 g /m^2 based MAC**						
MC-FludT.14/L Trial I	-0.26	0.21	152	168	21.0%	0.77 [0.51, 1.16]
**Subtotal (95% CI)**			**152**	**168**	**21.0%**	**0.77 [0.51, 1.16]**
Heterogeneity: Not applicable						
Test for overall effect: Z = 1.24 (P = 0.22)						
**Total (95% CI)**			**693**	**683**	**100.0%**	**1.00 [0.66, 1.50]**
Heterogeneity: Tau^2 = 0.16; Chi^2 = 16.75, df = 4 (P = 0.002); I^2 = 76%						
Test for overall effect: Z = 0.02 (P = 0.99)						
Test for subgroup differences: Chi^2 = 8.59, df = 2 (P = 0.01), I^2 = 76.7%						

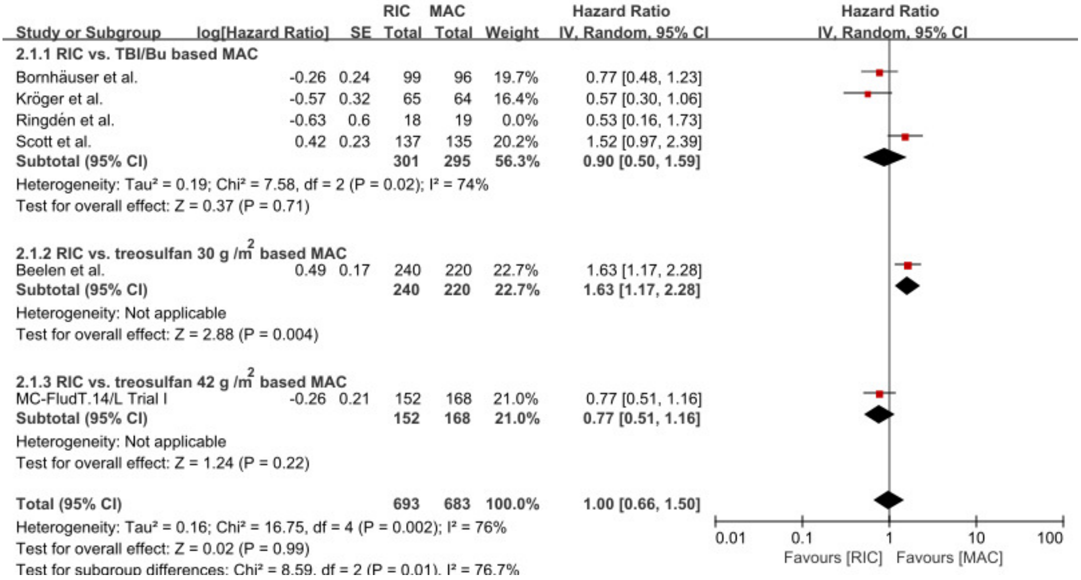

Cumulative incidence of relapse without Ringdén et al.

Study or Subgroup	log[Hazard Ratio]	SE	RIC Total	MAC Total	Weight	Hazard Ratio IV, Random, 95% CI
**2.2.1 RIC vs. TBI/Bu based MAC**						
Bornhäuser et al.	0.1	0.28	99	96	15.9%	1.11 [0.64, 1.91]
Kröger et al.	0.21	0.45	65	64	8.3%	1.23 [0.51, 2.98]
Ringdén et al.	-0.97	0.71	18	19	0.0%	0.38 [0.09, 1.52]
Scott et al.	0.72	0.22	137	135	20.4%	2.05 [1.33, 3.16]
**Subtotal (95% CI)**			**301**	**295**	**44.5%**	**1.50 [0.97, 2.31]**
Heterogeneity: Tau^2 = 0.06; Chi^2 = 3.35, df = 2 (P = 0.19); I^2 = 40%						
Test for overall effect: Z = 1.81 (P = 0.07)						
**2.2.2 RIC vs. treosulfan 30 g /m^2 based MAC**						
Beelen et al.	0.14	0.2	240	220	22.2%	1.15 [0.78, 1.70]
**Subtotal (95% CI)**			**240**	**220**	**22.2%**	**1.15 [0.78, 1.70]**
Heterogeneity: Not applicable						
Test for overall effect: Z = 0.70 (P = 0.48)						
**2.2.3 RIC vs. treosulfan 42 g /m^2 based MAC**						
MC-FludT.14/L Trial I	-0.01	0.09	152	168	33.2%	0.99 [0.83, 1.18]
**Subtotal (95% CI)**			**152**	**168**	**33.2%**	**0.99 [0.83, 1.18]**
Heterogeneity: Not applicable						
Test for overall effect: Z = 0.11 (P = 0.91)						
**Total (95% CI)**			**693**	**683**	**100.0%**	**1.23 [0.92, 1.64]**
Heterogeneity: Tau^2 = 0.06; Chi^2 = 9.53, df = 4 (P = 0.05); I^2 = 58%						
Test for overall effect: Z = 1.42 (P = 0.15)						
Test for subgroup differences: Chi^2 = 3.14, df = 2 (P = 0.21), I^2 = 36.2%						

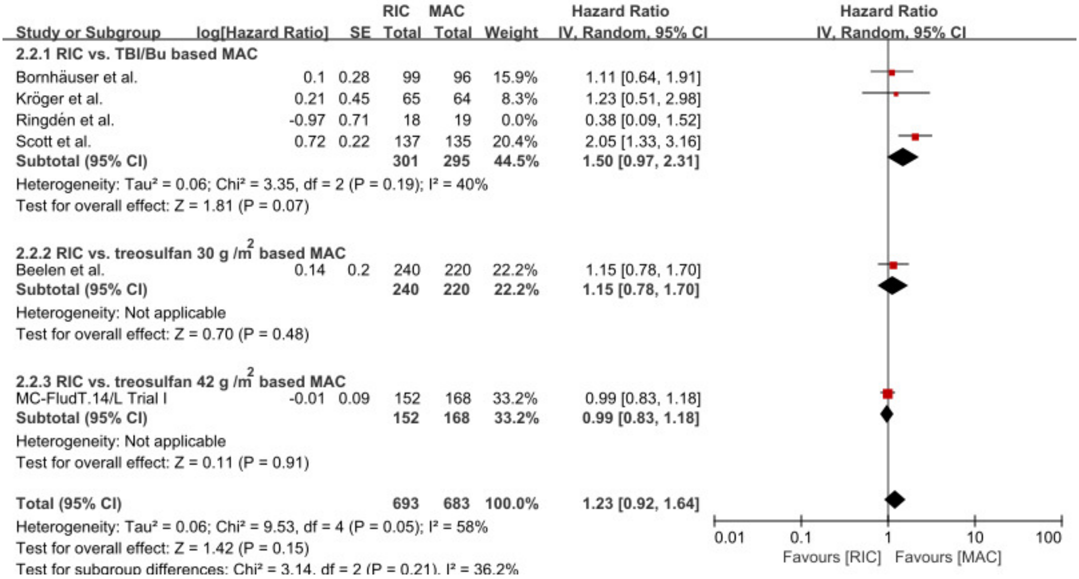

Non-relapse mortality without Ringdén et al.

Study or Subgroup	log[Hazard Ratio]	SE	RIC Total	MAC Total	Weight	Hazard Ratio IV, Fixed, 95% CI
**3.1.1 RIC vs. TBI/Bu based MAC**						
Bornhäuser et al.	-0.48	0.37	99	96	32.7%	0.62 [0.30, 1.28]
Kröger et al.	-0.38	0.36	65	64	34.6%	0.68 [0.34, 1.38]
Ringdén et al.	0.08	1	18	19	0.0%	1.08 [0.15, 7.69]
Scott et al.	-1.13	0.37	137	135	32.7%	0.32 [0.16, 0.67]
**Subtotal (95% CI)**			**301**	**295**	**100.0%**	**0.52 [0.34, 0.78]**
Heterogeneity: Chi^2 = 2.46, df = 2 (P = 0.29); I^2 = 19%						
Test for overall effect: Z = 3.11 (P = 0.002)						
**3.1.2 RIC vs. treosulfan 30 g /m^2 based MAC**						
Beelen et al.	0.51	0.25	240	220	100.0%	1.67 [1.02, 2.72]
**Subtotal (95% CI)**			**240**	**220**	**100.0%**	**1.67 [1.02, 2.72]**
Heterogeneity: Not applicable						
Test for overall effect: Z = 2.04 (P = 0.04)						
**3.1.3 RIC vs. treosulfan 42 g /m^2 based MAC**						
MC-FludT.14/L Trial I	-0.27	0.27	152	168	100.0%	0.76 [0.45, 1.30]
**Subtotal (95% CI)**			**152**	**168**	**100.0%**	**0.76 [0.45, 1.30]**
Heterogeneity: Not applicable						
Test for overall effect: Z = 1.00 (P = 0.32)						
Test for subgroup differences: Chi^2 = 12.82, df = 2 (P = 0.002), I^2 = 84.4%						

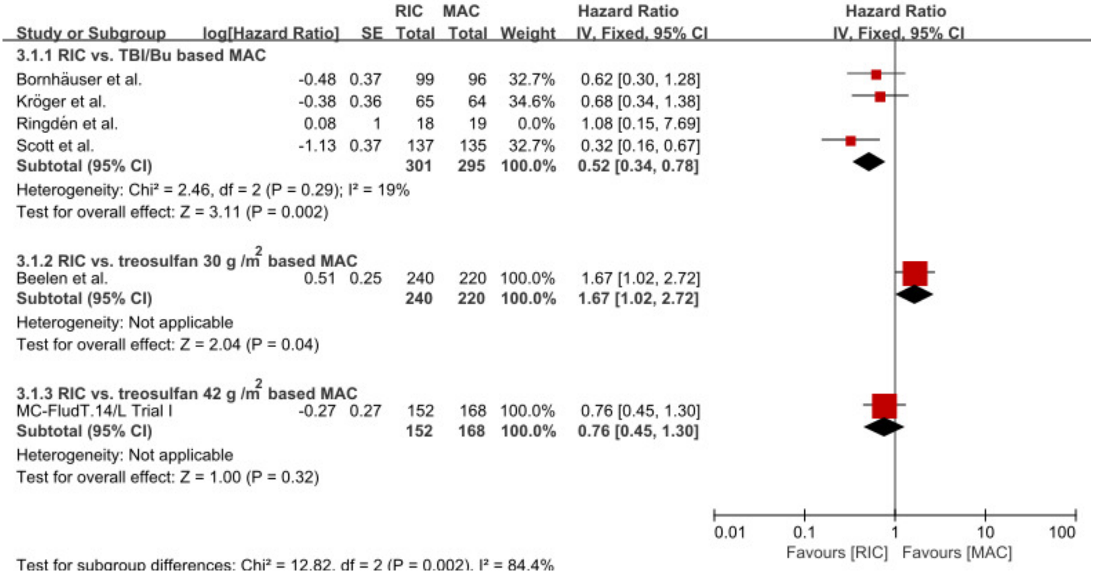

Supplement 9

Author(s):

Date: 2021-01-05

Question: Should RIC vs MAC be used for AML in CR and MDS?

Settings:

Bibliography: . reduced condition regimen for myeloid malignance. Cochrane Database of Systematic Reviews [Year], Issue [Issue].

Quality assessment							No of patients		Effect		Quality	Importance
No of studies	Design	Risk of bias	Inconsistency	Indirectness	Imprecision	Other considerations	RIC	MAC	Relative (95% CI)	Absolute		
**OS, CIR and LFS - OS (follow-up 0-60 months; assessed with: hazard ratio (HR))**												
6	randomised trials	no serious risk of bias	serious[1]	no serious indirectness	no serious imprecision	none	0/711 (0%)	0/702 (0%)	HR 1.07 (0.89 to 1.3)	-	⊕⊕⊕O MODERATE	CRITICAL
								0%		-		
**OS, CIR and LFS - CIR (follow-up 0-60 months; assessed with: hazard ratio (HR))**												
6	randomised trials	no serious risk of bias	serious[2]	no serious indirectness	no serious imprecision	none	0/711 (0%)	0/702 (0%)	HR 1.1 (0.95 to 1.27)	-	⊕⊕⊕O MODERATE	CRITICAL
								0%		-		
**OS, CIR and LFS - LFS (follow-up 0-60 months; assessed with: hazard ratio (HR))**												
3	randomised trials	no serious risk of bias	serious[3]	no serious indirectness	no serious imprecision	none	0/301 (0%)	0/295 (0%)	HR 1.18 (0.91 to 1.54)	-	⊕⊕⊕O MODERATE	CRITICAL
								0%		-		

[1] One study indicated RIC reduced OS while the other studies did not.

[2] One study suggested RIC increased RI while the other studies did not.

[3] One study showed RIC decreased LFS while the other studies did not.

Supplement 10

Author(s):

Date: 2021-01-05

Question: Should RIC vs MAC be used for AML in CR and MDS?

Settings:

Bibliography: . reduced condition regimen for myeloid malignance. Cochrane Database of Systematic Reviews [Year], Issue [Issue].

Quality assessment							No of patients		Effect		Quality	Importance
**No of studies**	**Design**	**Risk of bias**	**Inconsistency**	**Indirectness**	**Imprecision**	**Other considerations**	**RIC**	**MAC**	**Relative (95% CI)**	**Absolute**		
**NRM - RIC vs. TBI/Bu based MAC (follow-up 0-60 months; assessed with: hazard ratio (HR))**												
4	randomised trials	no serious risk of bias	no serious inconsistency	no serious indirectness	no serious imprecision	none	0/319 (0%)	0/314 (0%)	HR 0.53 (0.36 to 0.8)	-	⊕⊕⊕⊕ HIGH	CRITICAL
								0%		-		
**aGVHD - RIC vs. TBI/Bu based MAC (follow-up 0-60 months; assessed with: hazard ratio (HR))**												
4	randomised trials	no serious risk of bias	no serious inconsistency	no serious indirectness	no serious imprecision	none	0/309 (0%)	0/302 (0%)	HR 0.79 (0.6 to 1.03)	-	⊕⊕⊕⊕ HIGH	CRITICAL
								0%		-		
**cGVHD - RIC vs. TBI/Bu based MAC (follow-up 0-60 months; assessed with: hazard ratio (HR))**												
4	randomised trials	no serious risk of bias	serious[1]	no serious indirectness	no serious imprecision	none	0/309 (0%)	0/302 (0%)	HR 1.07 (0.68 to 1.69)	-	⊕⊕⊕O MODERATE	CRITICAL
								0%		-		

[1] No explanation was provided

Supplement 11

Overall survival without Beelen et al.

Study or Subgroup	log[Hazard Ratio]	SE	RIC Total	MAC Total	Weight	Hazard Ratio IV, Random, 95% CI
**2.1.1 RIC vs. TBI/Bu based MAC**						
Bornhäuser et al.	-0.26	0.24	99	96	23.6%	0.77 [0.48, 1.23]
Kröger et al.	-0.57	0.32	65	64	18.2%	0.57 [0.30, 1.06]
Ringdén et al.	-0.63	0.6	18	19	7.8%	0.53 [0.16, 1.73]
Scott et al.	0.42	0.23	137	135	24.4%	1.52 [0.97, 2.39]
**Subtotal (95% CI)**			**319**	**314**	**74.0%**	**0.84 [0.50, 1.40]**
Heterogeneity: $Tau^2 = 0.16; Chi^2 = 8.47, df = 3 (P = 0.04); I^2 = 65\%$						
Test for overall effect: Z = 0.67 (P = 0.50)						
**2.1.2 RIC vs. treosulfan 30 g /m^2 based MAC**						
Beelen et al.	0.49	0.17	240	220	0.0%	1.63 [1.17, 2.28]
**Subtotal (95% CI)**			**0**	**0**		**Not estimable**
Heterogeneity: Not applicable						
Test for overall effect: Not applicable						
**2.1.3 RIC vs. treosulfan 42 g /m^2 based MAC**						
MC-FludT.14/L Trial I	-0.26	0.21	152	168	26.0%	0.77 [0.51, 1.16]
**Subtotal (95% CI)**			**152**	**168**	**26.0%**	**0.77 [0.51, 1.16]**
Heterogeneity: Not applicable						
Test for overall effect: Z = 1.24 (P = 0.22)						
**Total (95% CI)**			**471**	**482**	**100.0%**	**0.84 [0.58, 1.21]**
Heterogeneity: $Tau^2 = 0.09; Chi^2 = 8.98, df = 4 (P = 0.06); I^2 = 55\%$						
Test for overall effect: Z = 0.95 (P = 0.34)						
Test for subgroup differences: $Chi^2 = 0.07, df = 1 (P = 0.80), I^2 = 0\%$						

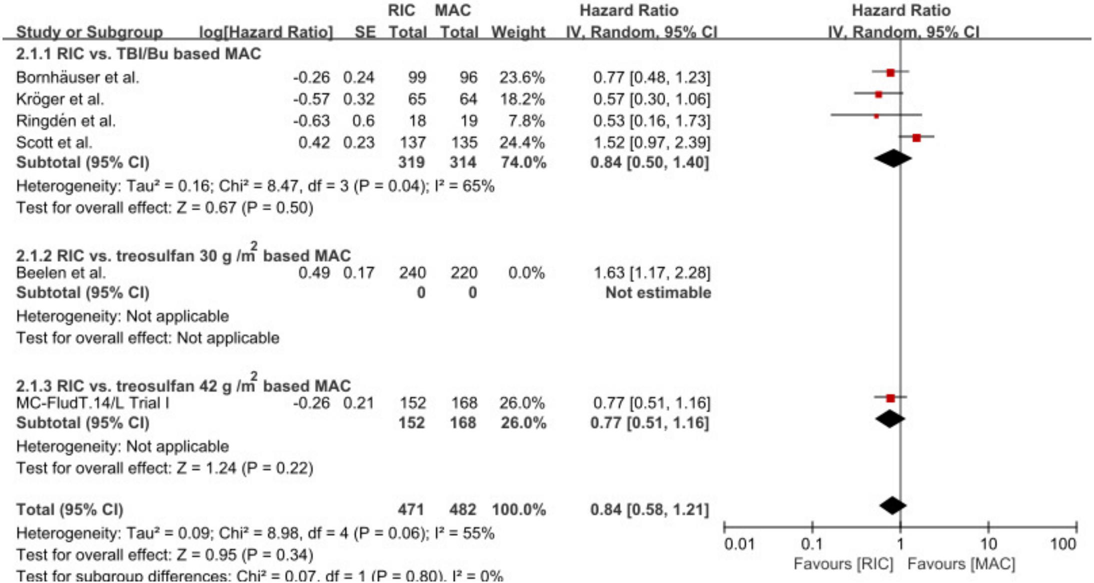

Supplement: Supplementary file 1 [file DataSheet_1.pdf]
